# SYVN1 modulates papillary thyroid carcinoma progression by destabilizing HMGB1

**DOI:** 10.1186/s13008-024-00121-1

**Published:** 2024-04-28

**Authors:** Fei Duan, Fanli Kong, Taifeng Jiang, Hongbing Liu

**Affiliations:** 1 Department of Otolaryngology Head and Neck Surgery, The Second Affiliated Hospital, Jiangxi Medical College, Nanchang University, Nanchang, 330006 China; 2 Department of Otolaryngology Head and Neck Surgery, Jiujiang First People’s Hospital, Jiujiang, 332600 China

**Keywords:** E3 ubiquitin ligase SYVN1, HMGB1, Papillary thyroid cancer

## Abstract

E3 ubiquitin ligase synoviolin (SYVN1) has been reported to participate in many human cancers. This study aimed to investigate SYVN1’s roles and molecular pathways in papillary thyroid cancer (PTC). The functions of SYVN1 in PTC were further analyzed using gain- and loss-of-function methods and numerous investigations in cellular function and molecular biology. The findings demonstrated that the overexpression of SYVN1 markedly suppressed the proliferation, migration, and invasion of PTC cell lines (NPA87 and TPC-1). We found that SYVN1 interacted with HMGB1 and promoted its ubiquitination and degradation. In addition, SYVN1 effectively impairs cell proliferation, migration, invasion, and the formation of tumor xenografts in mice models. However, this effect may be partly reversed by overexpressing HMGB1. Thus, SYVN1 may inhibit the proliferation, migration, and invasion of PTC cells by disrupting HMGB1. Consequently, SYVN1 might be considered a promising therapeutic target for PTC.

**Supplementary Information:**

The online version contains supplementary material available at 10.1186/s13008-024-00121-1.

## Introduction

Thyroid cancer (TC) is a prevalent kind of endocrine malignancy that is seeing a significant rise in occurrence globally. It has been characterized as a sort of epidemic overdiagnosis, a phenomenon in which the incidence of malignant tumors increases without an increase in mortality, known as the “overdiagnosis” of malignant tumors [1]. Furthermore, there have been reports of four histological kinds of TC: papillary, follicular, medullary, and poorly differentiated. These types represent the significant diversity seen in TC [7]. Significantly, this variability encompasses variations in tissue pathology and disparities in several genetic and epigenetic modifications, thus posing greater diagnostic and prognostic challenges for patients with high-risk TC [6]. Papillary TC (PTC) is a major subtype of thyroid cancer with an increasing incidence worldwide and is prone to early neck lymph node metastasis, making treatment complex [12]. Therefore, an in-depth study of the pathogenesis of PTC and a search for potential targets are of great significance for clinical diagnosis and treatment.

Synoviolin 1 (SYVN1), also known as Hrd1, is an evolutionarily conserved endoplasmic reticulum E3 ligase that plays an important role in regulating a variety of physiological processes, including ER stress, chronic inflammation, vascular growth, oxidative stress, and apoptosis, as well as in promoting the degradation of misfolded proteins during ubiquitin-proteasome-dependent endoplasmic reticulum-associated degradation [16]. SYVN1 targets multiple substrates and is involved in various cancers by regulating ubiquitin-proteasome-dependent degradation of key molecules such as p53 and Sirtuin 2 [9, 13]. Thus, the role of SYVN1 in tumourigenesis and cancer development is paradoxical and dependent on the cellular environment and microenvironment. Previously, ubiquitinomics analysis showed that SYVN1 is overexpressed in hepatocellular carcinoma (HCC) and promotes tumourigenesis and metastasis. However, the role of SYVN1 in PTC is unknown.

This study centered on the differential expression of SYVN1 in PTC and its effect on the malignant behavior of PTC cells. We experimentally discovered that SYVN1 inhibited cell growth, migration, and invasion of PTC cells by disrupting HMGB1 in PTC cells. These results suggest that SYVN1 is a potential therapeutic target for PTC.

## Results

### SYVN1 is significantly decreased in PTC tissues and cells

**Fig. 1:**
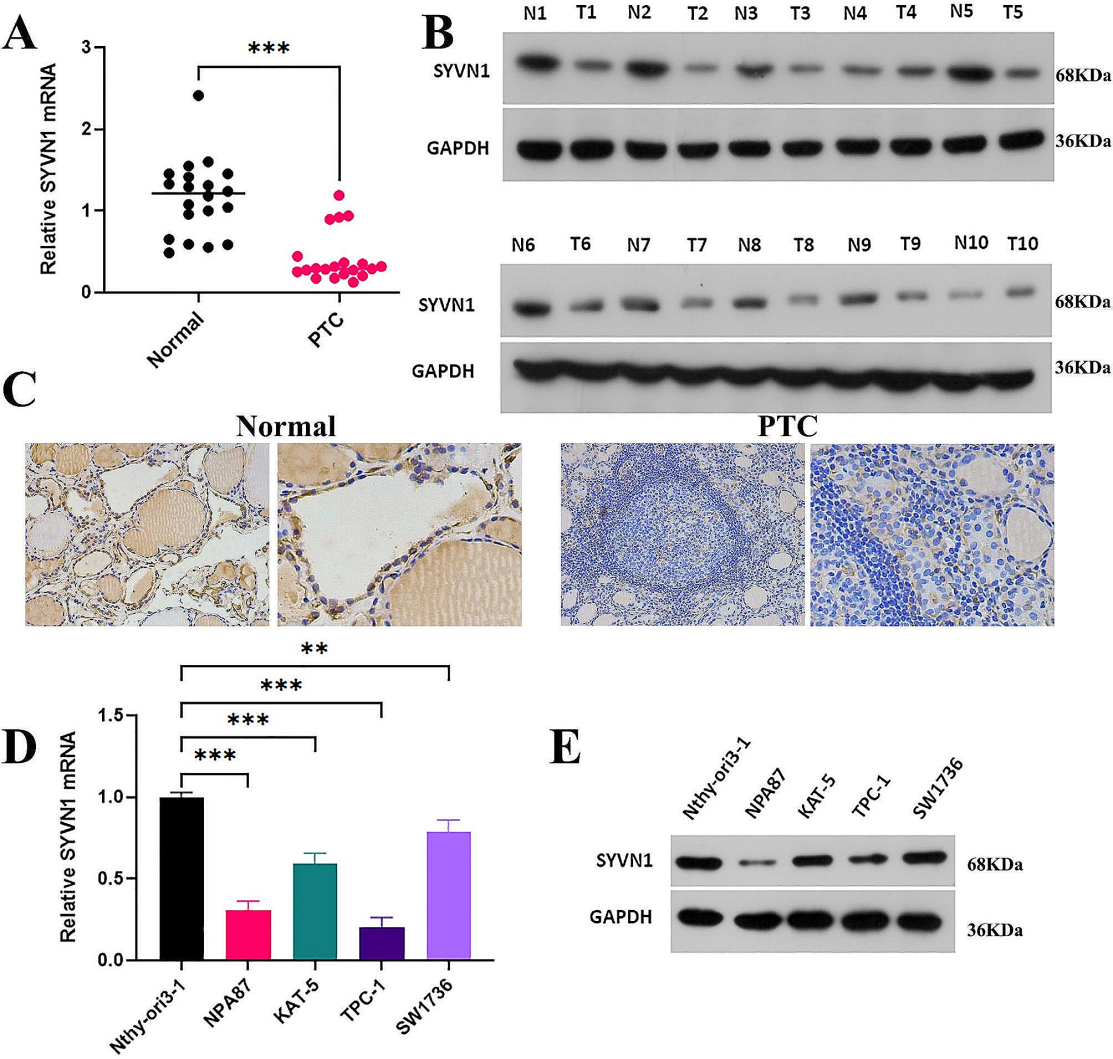
SYVN1 is significantly decreased in PTC tissues and cells. (**A**) RT-PCR and (**E**) WB were used to measure the mRNA and protein expression levels of SYVN1 on 10 paired human PTC samples and non-tumor thyroid tissues. (**C**) Representative IHC staining of SYVN1 in PTC tissues. (**D**) RT-PCR quantified SYVN1 mRNAs. (**E**) Differential expression of SYVN1 protein was compared in PTC cells

To study the expression changes of SYVN1 in PTC, we analyzed the SYVN1 mRNA and protein levels in 10 pairs of PTC tissues via RT-PCR and WB. The findings showed that the mRNA and protein expression of SYVN1 was downregulated in the PTC tissues (Fig. 1A, B). In addition, IHC studies of PTC samples revealed decreased SYVN1 protein levels in PTC tissues (Fig. 1C). Meanwhile, compared to Nthy-ori3–1, SYVN1 was remarkably reduced in PTC cell lines NPA87, KAT-5, TPC-1, and SW1736 (especially in NPA87 and TPC-1), which was chosen for the following experiments (Fig. 1D, E).

### SYVN1 suppresses PTC cell proliferation

**Fig. 2:**
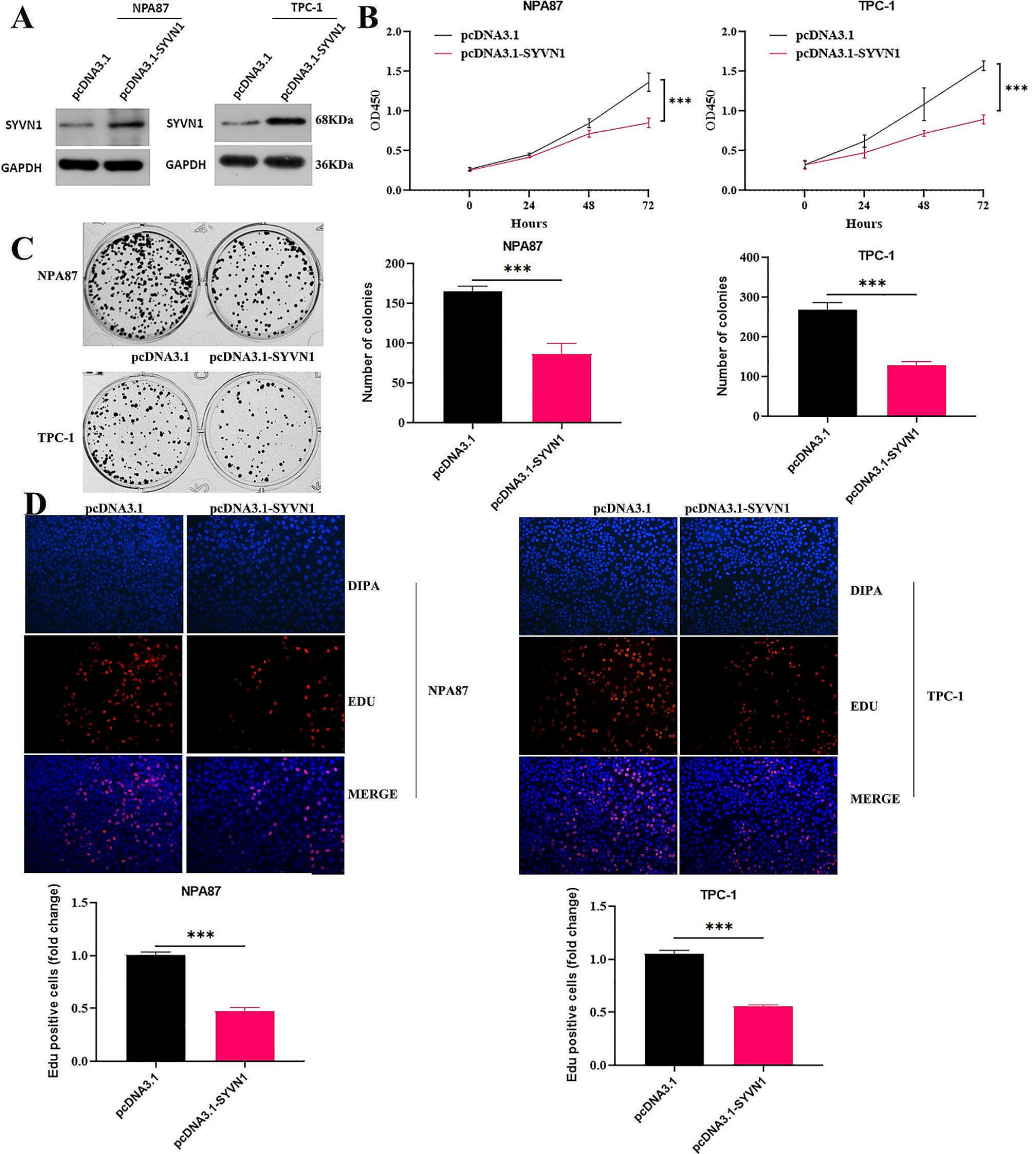
SYVN1 inhibits PTC cell growth. (**A**) WB detection for SYVN1 in PTC cells after pcDNA3.1-SYVN1. Cell proliferation was detected using (**B**) CCK-8, (**C**) colony formation, and (**D**) EDU assays after transfection of pcDNA3.1-SYVN1 into NPA87 and TPC-1 cells

The first finding is that SYVN1 expression in cells was elevated after pcDNA3.1-SYVN1 transfection, as shown in Fig. 2A. Results from the CCK-8 test (Fig. 2B), clone formation (Fig. 2C), and EdU assay (Fig. 2D) showed that the pcDNA3.1-SYVN1 group decreased PTC cell growth when compared to the pcDNA3.1 group.

### SYVN1 inhibits migration and invasion in PTC cells

**Fig. 3:**
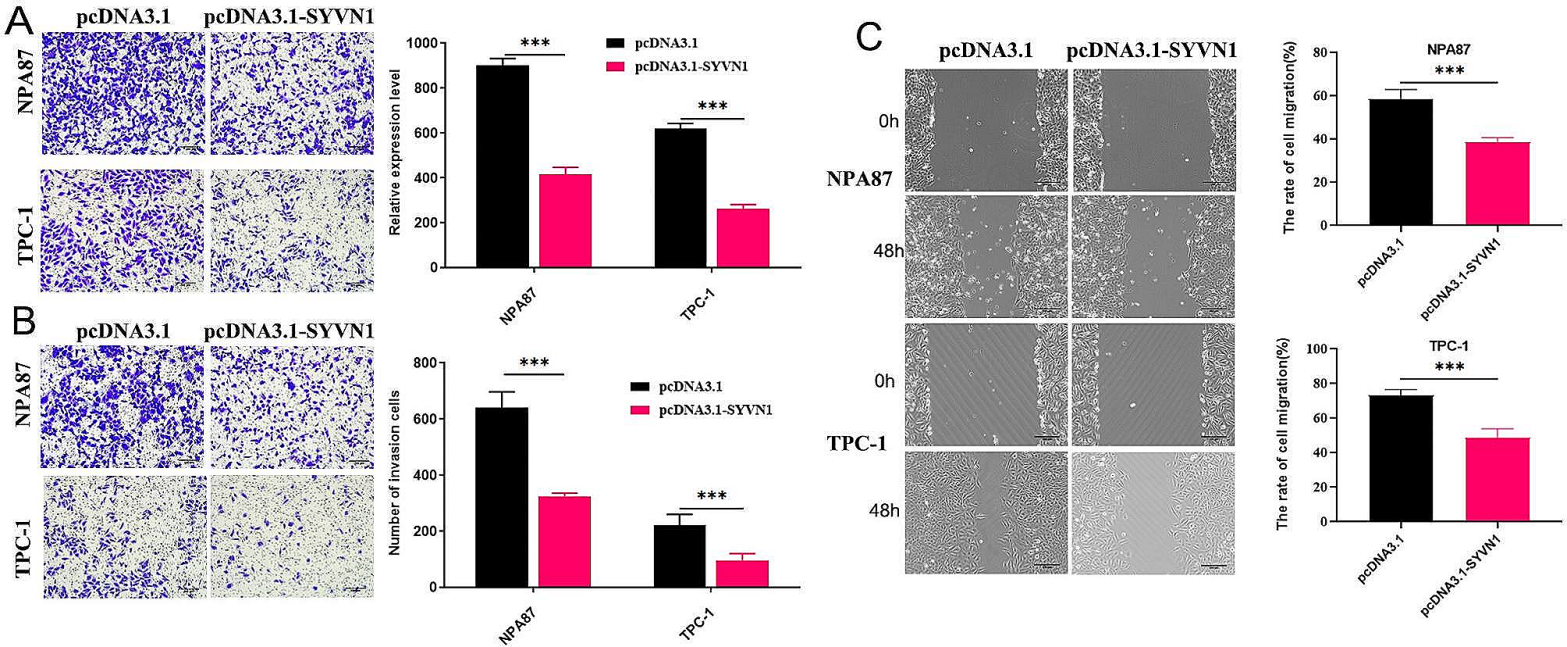
SYVN1 inhibits migration and invasion in PTC cells. (**A**, **B**) Transwell assay examined the migratory and invasive capacities of PTC cells transfected with pcDNA3.1-SYVN1 or pcDNA3.1. (**C**) Wound healing was used to evaluate the migratory capabilities of PTC cells transfected with pcDNA3.1-SYVN1

Transwell test results showed overexpressing SYVN1 reduced NPA87 and TPC-1 cell motility and invasion capabilities (Fig. 3**A**, **B**). The experiment on healing scratch wounds similarly showed that the increased expression of SYVN1 reduced the ability of PTC cells to migrate (Fig. 3**C**).

### SYVN1 regulates HMGB1 protein degradation via ubiquitination

**Fig. 4:**
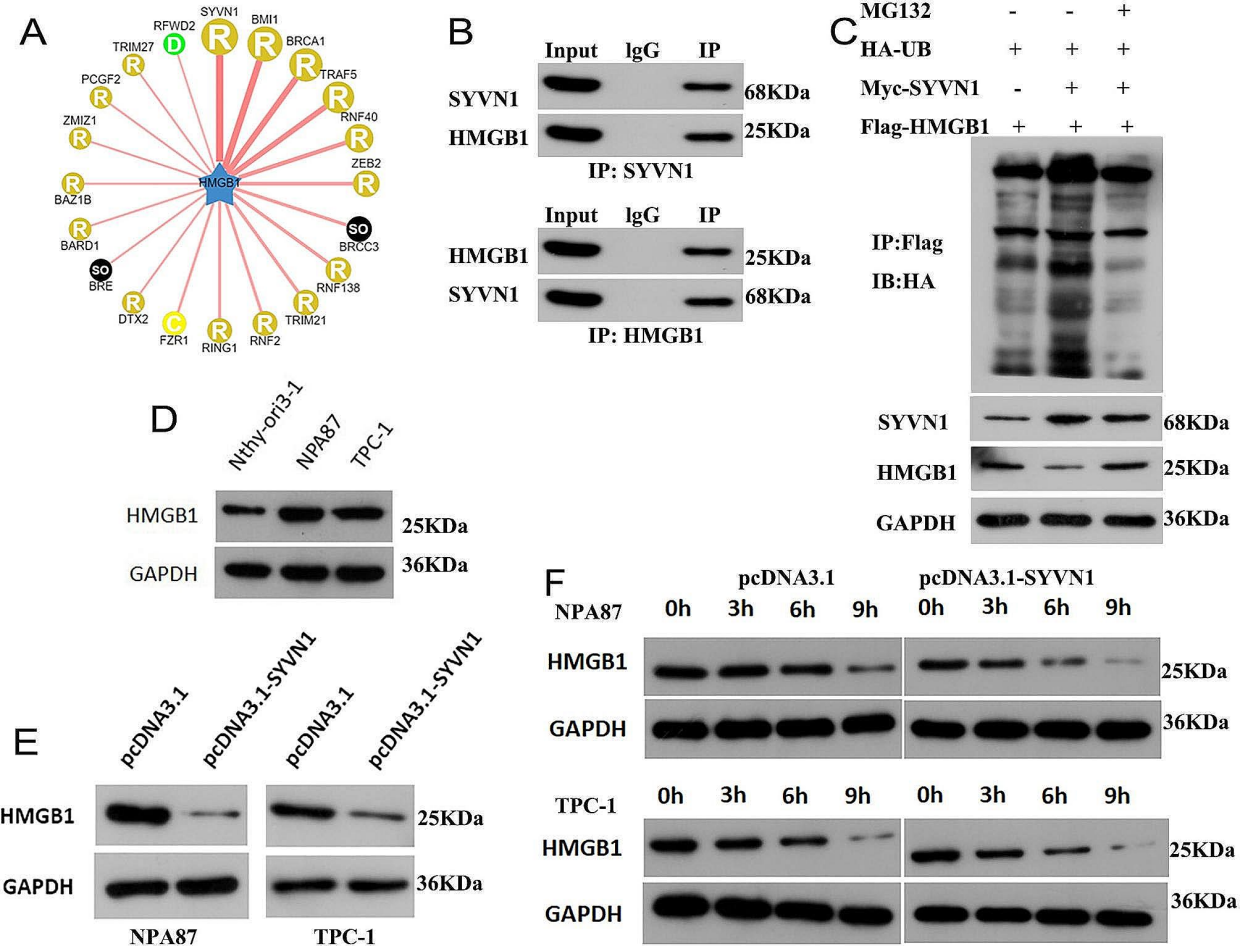
SYVN1 regulates HMGB1 protein degradation via ubiquitination. (**A**) Prediction of potential E3 ligase of HMGB1. (**B**) Co-IP analyzed the protein interaction between SYVN1 and HMGB1 proteins in NPA87 cells. (**C**) Effect of SYVN1 on HMGB1ubiquitylation in NPA87 cells. (**D**) WB showed the expression of HMGB1 in PTC cells. (**E**) WB shows the interference effect of the pcDNA3.1-SYVN1 interference on the HMGB1 protein in NPA87 and TPC-1 cells. (**F**) The half-life of HMGB1 in different groups

To elucidate the molecular mechanisms by which SYVN1 regulates the development of PTC, we used the UbiBrowser database (http://ubibrowser.bio-it.cn) and found that HMGB1 is a candidate ubiquitination substrate for SYVN1 (Fig. 4A). Therefore, a Co-IP assay was performed to verify this prediction, and the results demonstrated that HMGB1 interacted with SYVN1 in PTC cells (Fig. 4B). Then, the effect of SYVN1 on the ubiquitination of HMGB1 was studied. We found that the ubiquitination of HMGB1 increased when overexpressing SYVN1, and inhibiting ubiquitination degradation with MG132 restored HMGB1 protein expression after SYVN1 overexpression (Fig. 4C). We also detected the protein levels of HMGB1 in PTC cells. Higher levels of HMGB1 were observed in NPA87 and TPC-1 cells (Fig. 4D). The next step was to determine how SYVN1 affected HMGB1 expression. Overexpression of SYVN1 gave rise to decreased HMGB1 protein levels in PTC cells (Fig. 4E). The cells were exposed to a concentration of 100ug/ml CHX to suppress the production of proteins. Our results show that SYVN1 affects HMGB1 degradation in PTC cells since overexpressing SYVN1 in these cells caused a reduction in the half-life of HMGB1 (Fig. 4F).

### Overexpression of HMGB1 reverses cellular migration and invasion induced by SYVN1 in PTC cells

**Fig. 5:**
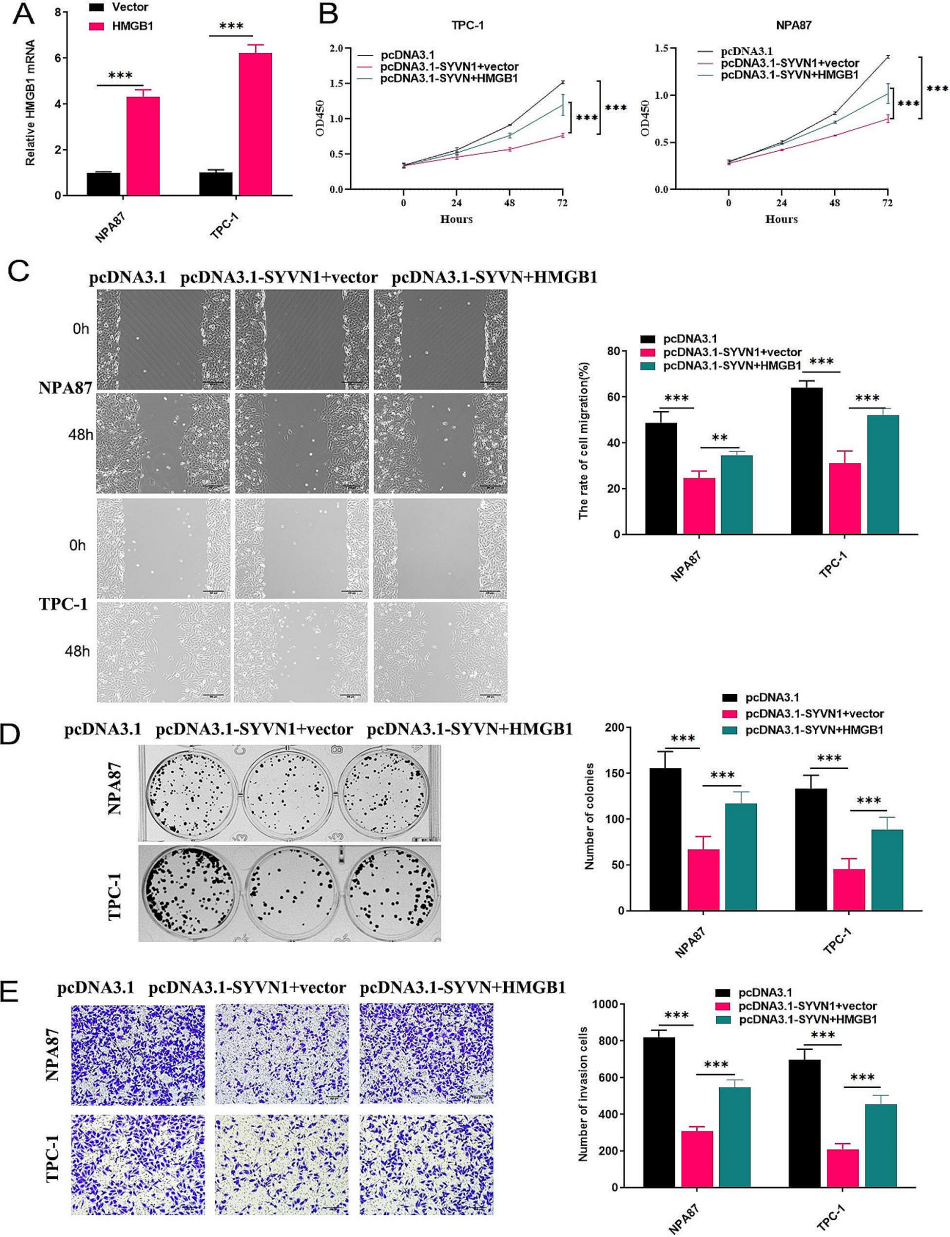
Overexpression of HMGB1 reverses cellular migration and invasion induced by SYVN1 in PTC cells. (**A**) The protein levels of HMGB1 levels were detected by western blot. (**B**) Cell viability. **(C**) Wound healing was used to evaluate migratory capabilities. (**D**) Colony formation assay. (**E**) Numbers of invasion cells in different groups

RT-qPCR assay results verified the successful upregulation of HMGB1 in cells after overexpression of HMGB1 (Fig. 5A). The findings showed that overexpressing SYVN1 in TPC-1 and NPA87 cells inhibited cell proliferation, wound healing, and the number of invasion cells. Further study by upregulation of HMGB1 reverses the effects of SYVN1 overexpression on proliferation, migration, and invasion of PTC cells (Fig. 5B-E). This data further proved that SYVN1 regulated HMGB1 expression to inhibit PTC cell proliferation, migration, and invasion.

### Overexpression of HMGB1 reverses PTC cell growth induced by SYVN1 in vivo

**Fig. 6:**
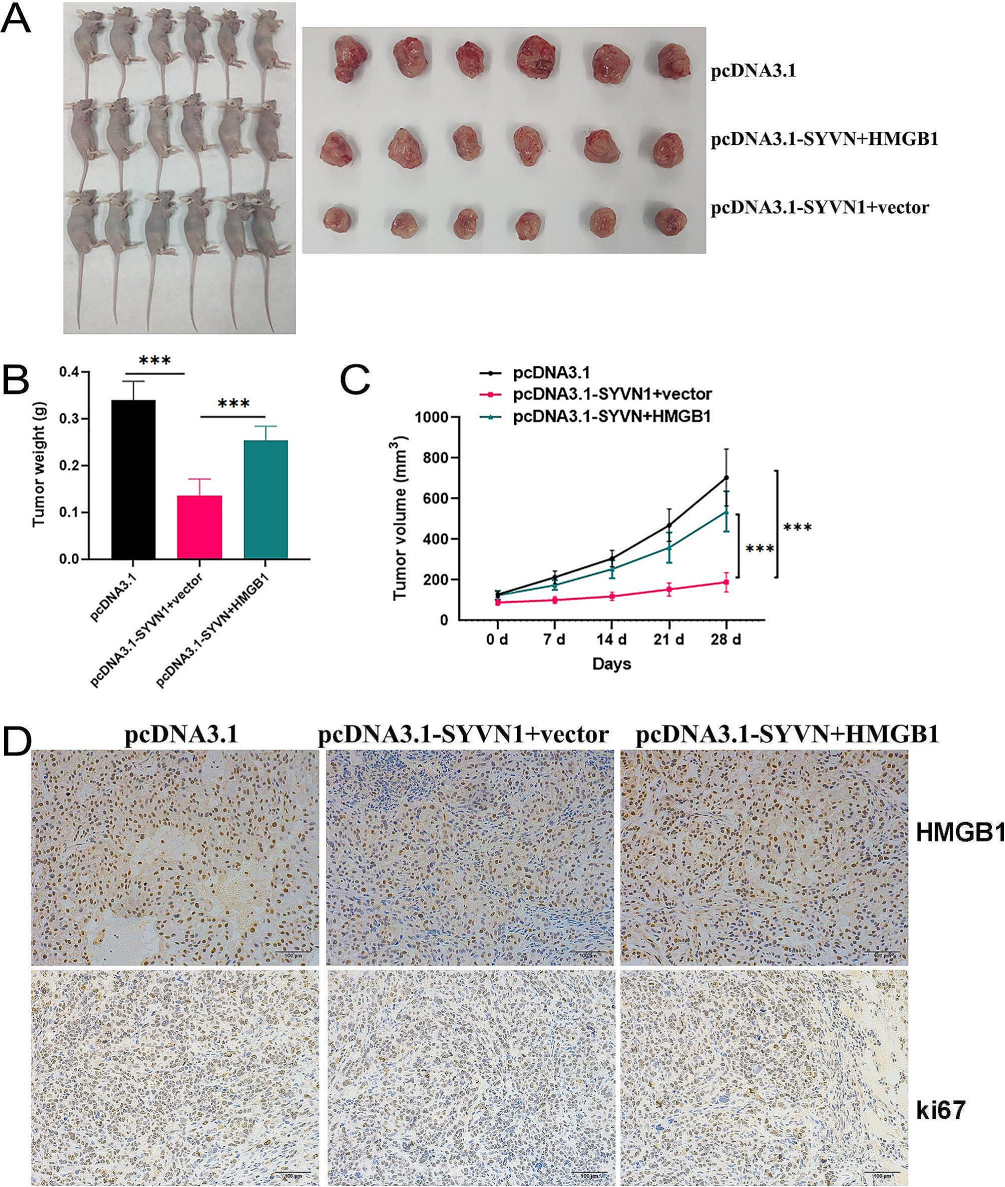
Overexpression of HMGB1 reverses PTC cell growth induced by SYVN1 in vivo. (**A**-**C**) Stable pcDNA3.1, pcDNA3.1-SYVN1 + vector, and pcDNA3.1-SYVN + HMGB1 transfected TPC-1 cells were injected subcutaneously into nude mice, and tumor lesions, volume, and weight were measured. (**D**) Representative IHC staining micrographs of Ki-67 and HMGB1 in tumor xenografts were conducted

We further used in vivo experiments to certify the results of in vitro experiments. After 28 days, all the mice were killed. As shown in Fig. 6A, the corresponding tumors were excised. The weight (Fig. 6B) and volume (Fig. 6C) of tumors were smaller in the pcDNA3.1-SYVN1 + vector group than in the pcDNA3.1 group, while this effect was abolished by HMGB1 overexpression. According to IHC results, ki67 and HMGB1 proteins were decreased by pcDNA3.1-SYVN1, while HMGB1 overexpression by co-transfection of pcDNA3.1-SYVN1 abolished all these abnormalities (Fig. 6D).

## Discussion

The incidence of PTC has shown a significant increase during the last several decades. While individuals with PTC typically have a favorable prognosis, the main characteristics that contribute to a bad prognosis are tumor invasiveness and metastasis. Hence, it is essential to clarify the molecular pathways that drive the course of PTC illness in order to pinpoint novel treatment targets and devise more potent therapeutic regimens for PTC.

The current investigation demonstrated that PTC cell lines and tissues exhibited downregulation of SYVN1. Our results also showed that overexpression of SYVN1 could suppress proliferation, migration, and invasion in PTC cell lines and inhibit tumorigenesis in vivo, indicating that SYVN1 plays a tumor-suppressive role in PTC. A previous study found that SYVN1 inhibits triple-negative breast tumourigenesis by ubiquitinating CPT2 and vimentin [4, 5]. In contrast to its tumor-suppressive activity, SYVN1 exerts tumor-promoting effects through ubiquitination and degradation of tumor-suppressors in different cancers, including colon cancer [11], prostate cancer [3], lung cancer [2], and hepatocellular carcinoma [8]. SYVN1 promotes non-small cell lung cancer metastasis by blocking autophagy-mediated MIEN1 degradation [15]. However, the molecular mechanism of SYVN1 in cancer progression is not fully clarified.

This work used bioinformatics analysis and immunoprecipitation tests to establish the interaction between the deubiquitinase SYVN1 and HMGB1. Through the destabilization of HMGB1 in PTC cells, the overexpression of SYVN1 has the impact of inhibiting proliferation, migration, and invasion. Previous studies also confirmed that using bioinformatics analysis, SYVN1 is the E3 ligase of HMGB1 [14]. A recent study found USP13, which belongs to the USP family, was identified as an E3 ligase of HMGB1 using LC–MS/MS, immunoprecipitation, and proximity ligation studies for HMGB1 ubiquitination [10]. HMGB1, one of the highly conserved nuclear proteins, is up-regulated in thyroid cancer and associated with clinicopathologic features. Our study found overexpression of HMGB1 reverses cellular migration and invasion induced by SYVN1 in PTC cells. One potential limitation of our study is the relatively small number of participants, and the correlation between the abundance of SYVN1 in examined tissues and patients’ overall survival should also be studied.

The findings of our study indicate that SYVN1 exhibits a substantial decrease in PTC and enhances the proliferation, migration, and invasion of PTC by destabilizing HMGB1. SYVN1 offers a theoretical foundation for the management of PTC and exhibits potential as a promising target for therapeutic interventions.

## Materials and methods

### Sample collection

Ten pairs of PTC adjacent normal tissues were provided by Jiujiang First People’s Hospital from July 2023 to September 2023. The tissue samples obtained from PTC patients were frozen in liquid nitrogen and then preserved at a temperature of -80 °C. All PTC patients involved in this study have signed informed consent to obtain their samples.

### Cell culture and cell transfection

Our research used human PTC cell lines (NPA87, KAT-5, TPC-1, SW1736) and a normal thyroid follicular cell line (Nthy-ori3–1). The cells were cultivated in Eagle’s Minimal Essential Media (MEM) with 10% fetal bovine serum (FBS; Hyclone) at 37 °C and a carbon dioxide concentration of 5%. NPA87 and TPC-1 cells were transfected with two different vector constructs (pcDNA3.1; pcDNA3.1- SYVN1) using Lipofectamine 3000 transfection reagent (Invitrogen, USA), following the manufacturer’s instructions.

### Cell proliferation assays

Transfected cells were seeded in 96-well plates (5 × 10^3^ cells/well). The proliferation of NPA87 and TPC-1 cells was assessed in each group using the CCK-8 assay (Beyotime Institute of Biotechnology) following the manufacturer’s protocols. The absorbance of each well was measured at 450 nm using a microplate reader.

### 5-Ethynyl-20-deoxyuridine (EdU) assay

The cells were transfected and then exposed to a concentration of 10 µM EdU (EdU Staining Proliferation Kit, Abcam) for an extra 24 h of incubation. The proliferation rate was calculated with EdU (red)-stained cells number/DAPI (blue)-stained cells x100%.

### Wound-healing assay

Transfected cells were cultured in 6-well plates to full confluence. Cell monolayers were scraped using a 200 µL pipette tip. Cell movement was observed at 0 and 48 h, using an Olympus microscope manufactured by Olympus.

### Transwell assay

The invasion capacity of NPA87 or TPC-1 cells was evaluated using Matrigel-Coated Transwell Chambers manufactured by BD Bioscience. The upper chamber was inoculated with cells. Following an overnight incubation period, the cells in the lower chamber were subjected to fixation using a 4% paraformaldehyde solution. Subsequently, staining was performed using a 0.1% crystal violet solution. The specimens were enumerated using a microscope manufactured by Olympus.

### Co-immunoprecipitation (Co-IP)

The cells were disrupted using IP lysis buffer (Beyotime) at 4 °C for 30 min. Subsequently, the mixture was centrifuged at a force of 13,000 g. During each immunoprecipitation, the liquid portion of the sample was mixed with Protein A/G agarose beads (Santa Cruz), which were used to separate and collect the target protein. After washing, the beads were gathered using centrifugation and combined with a loading buffer. Subsequently, protein separation was carried out using SDS-PAGE, followed by immunoblotting.

### Immunohistochemical (IHC) analysis

Tissues were fixed, embedded, sectioned, and removed. Nonspecific antibody binding sites in sections were blocked with serum-free protein block buffer (DAKO) for 30 min. The sections were then incubated with anti-SYVN1 antibody (1: 200, Abcam).

### Xenograft model

A tumor xenograft experiment was conducted to investigate the function of DLGAP5 in vivo. Stable transfection of pcDNA3.1, pcDNA3.1-SYVN1 + vector, pcDNA3.1-SYVN + HMGB1 in TPC-1 cells (100 µL, ∼ 5.0 × 10^6 cells) was subcutaneously injected into 4-week-old male nude mice. The tumor size was measured after a 10-day observation period using the formula (Length × Width^2) × 0.5. After 28 days, the mice were euthanized, and the weight of the xenograft tumors was determined.

### Quantitative real‑time PCR (RT-PCR) and Western blotting (WB)

Total RNA was extracted from tissues or cells using Trizol reagent and reverse transcription into cDNA using a kit (Takara). RT-PCR was performed using suitable primers. Total proteins were extracted from tissues or cells for WB analysis. Antibodies used included SYVN1 (1:1000; Abcam), HMGB1 (1:1000; Proteintech) and GAPDH (1:5000; Proteintech). HRP-conjugated goat anti-rabbit (1:10000; Proteintech) antibodies were used as secondary antibodies.

### Statistical analysis

Statisticians employed GraphPrism version 9.0. Each value represents the mean ± standard deviation of three independent experiments. Comparing groups required a one-way ANOVA with Tukey’s post hoc test or a Student’s t-test. Values of *P < 0.05; **P < 0.01; ***P < 0.001 ere considered significant.

### Electronic supplementary material

Below is the link to the electronic supplementary material.

Supplementary Material 1

## Data Availability

All data generated or analyzed during this study are included in this article.
