# Supplementary figures and images for: SYVN1 modulates papillary thyroid carcinoma progression by destabilizing HMGB1
Source: Cell Div. 2024 Apr 28;19:15. doi: 10.1186/s13008-024-00121-1 (PMC11057142; doi:10.1186/s13008-024-00121-1)

**Uncropped gels:**

For Figure 1B:


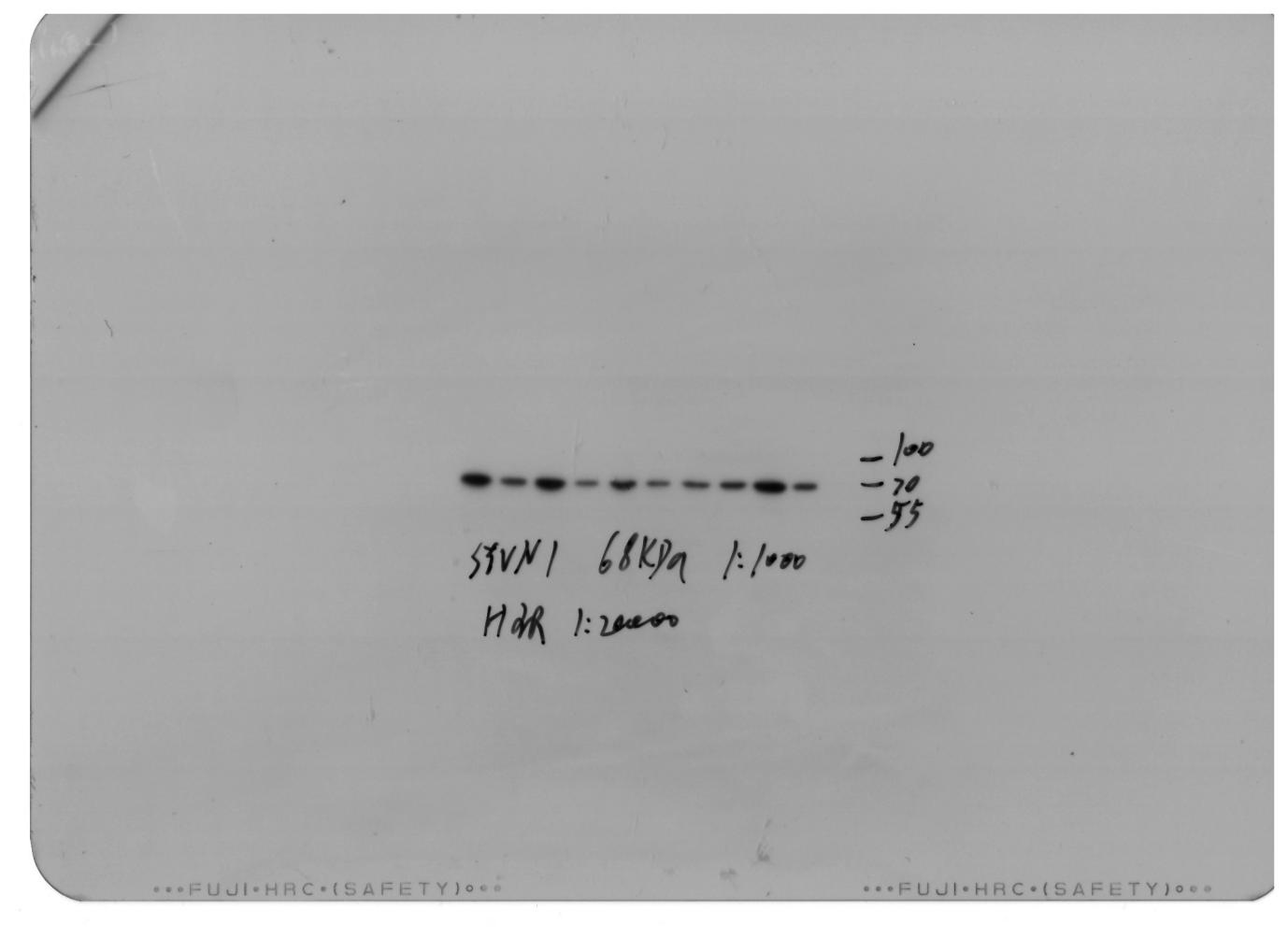


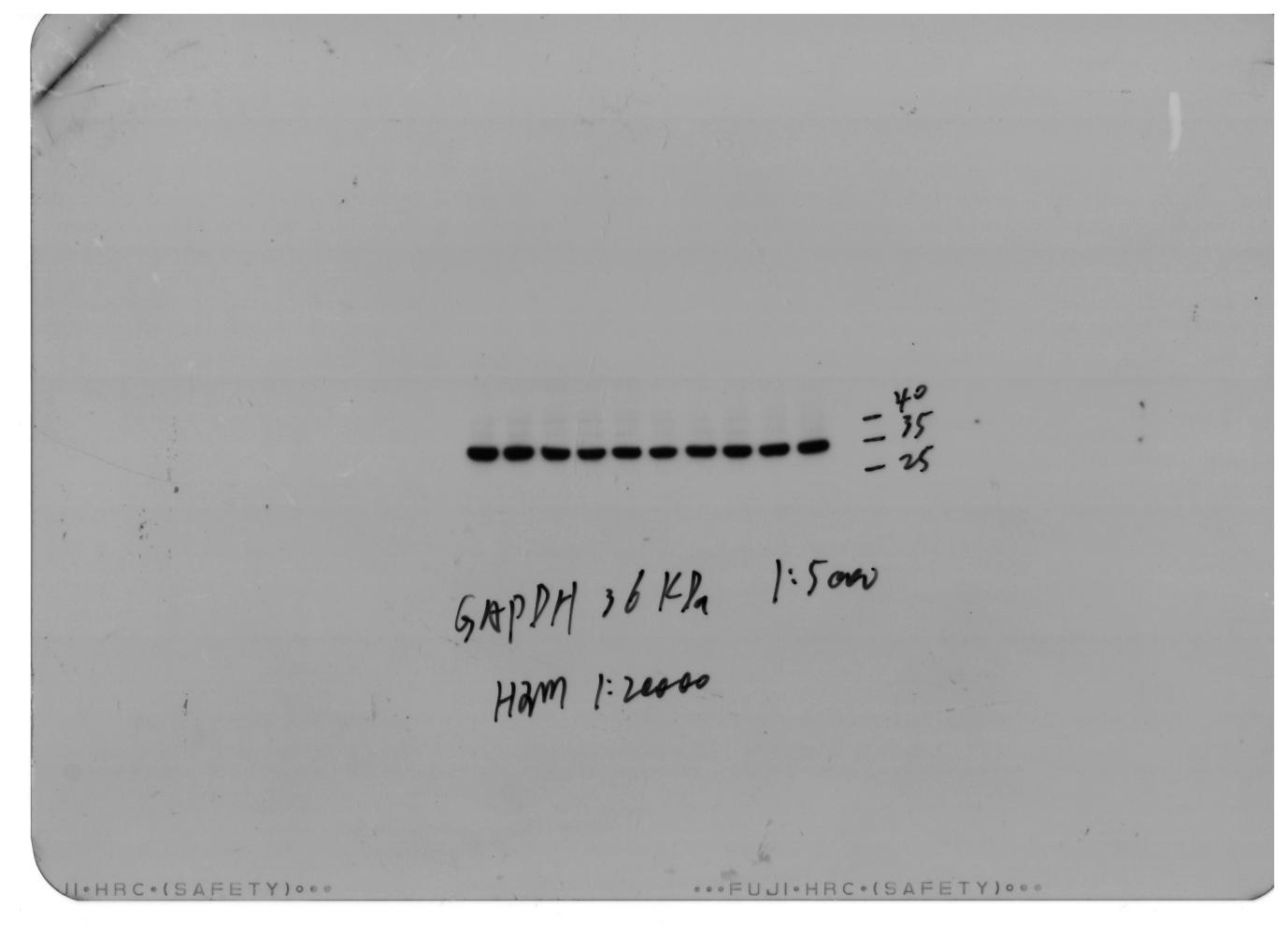


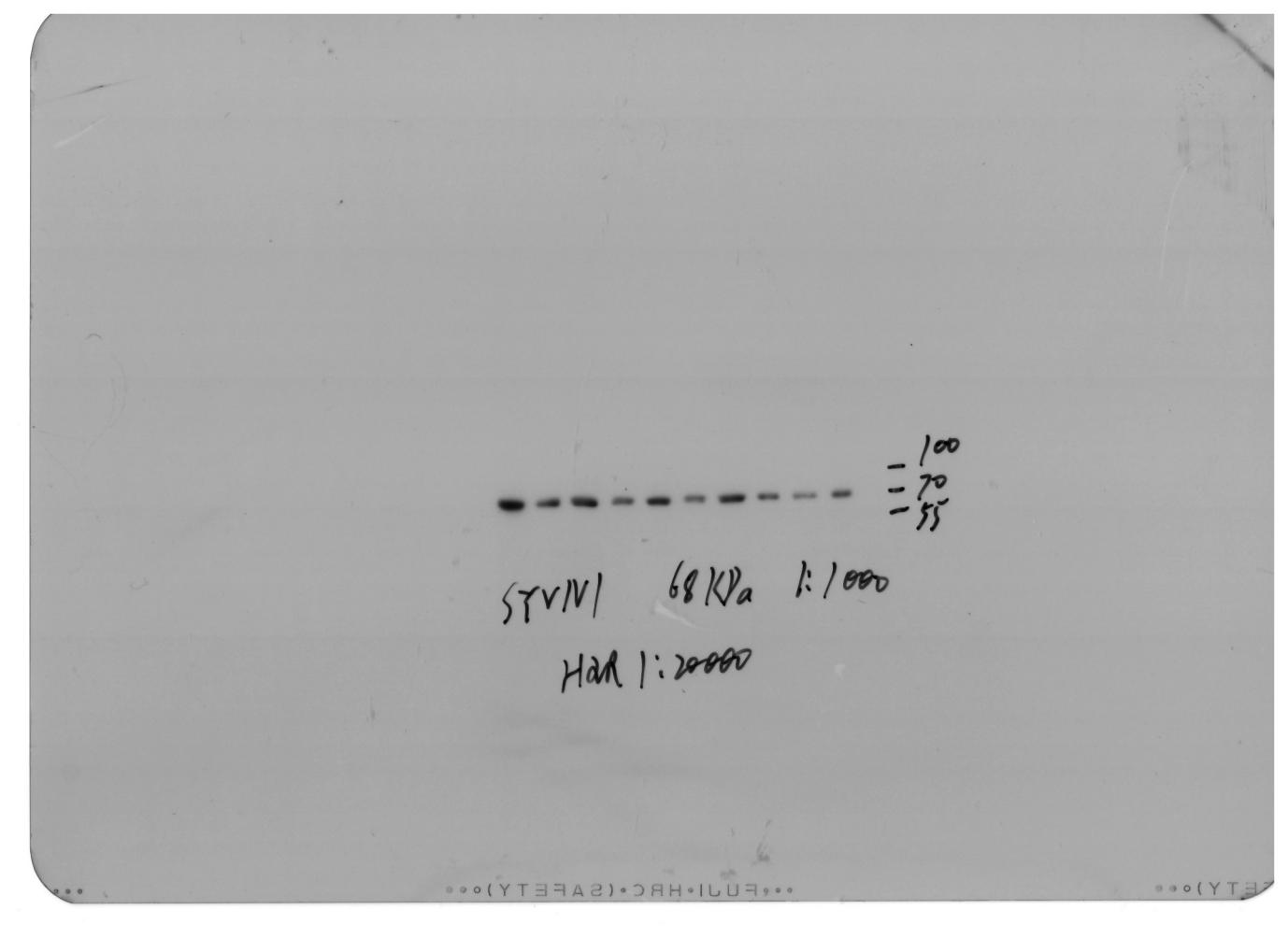


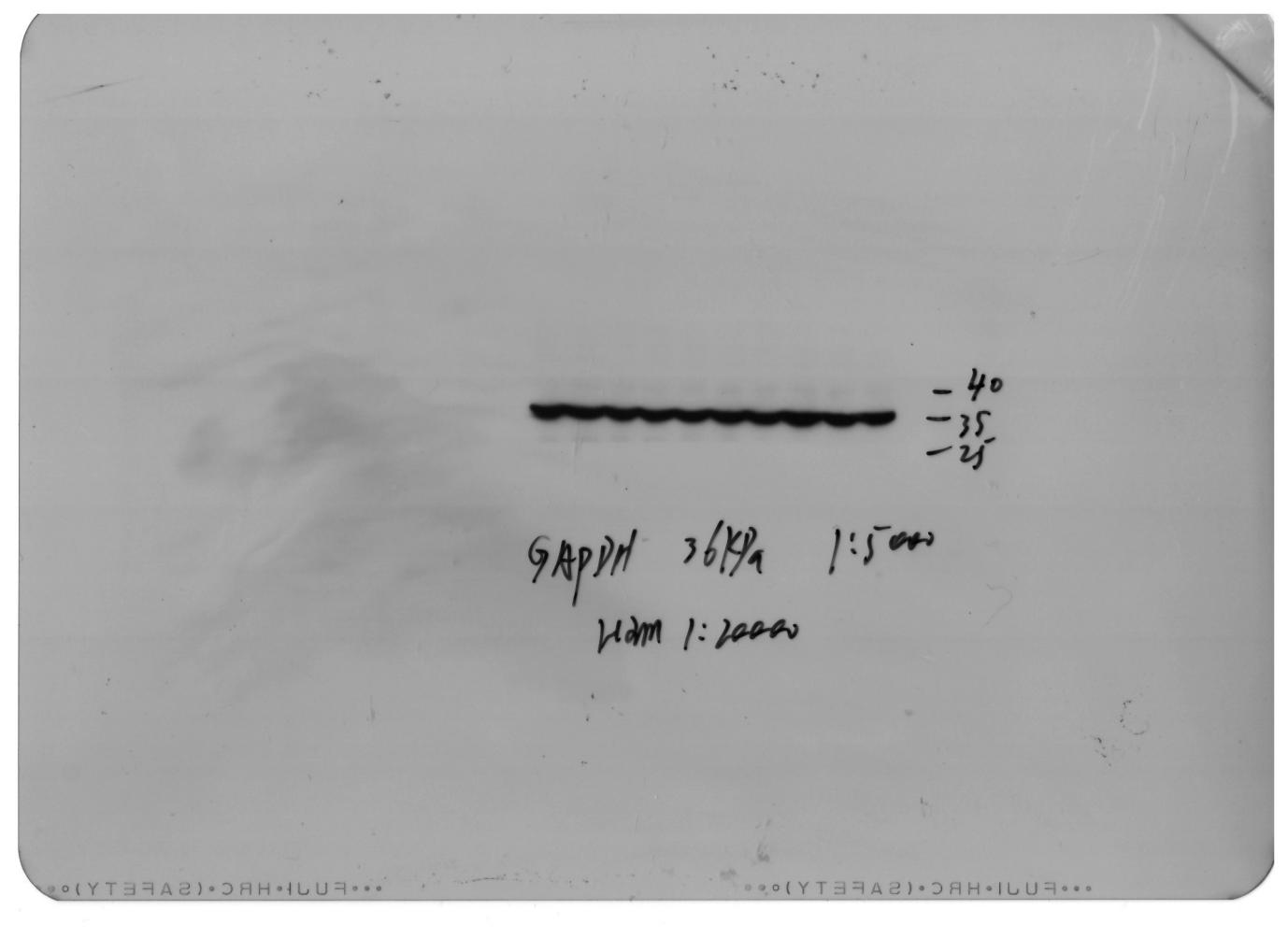


Figure 1E


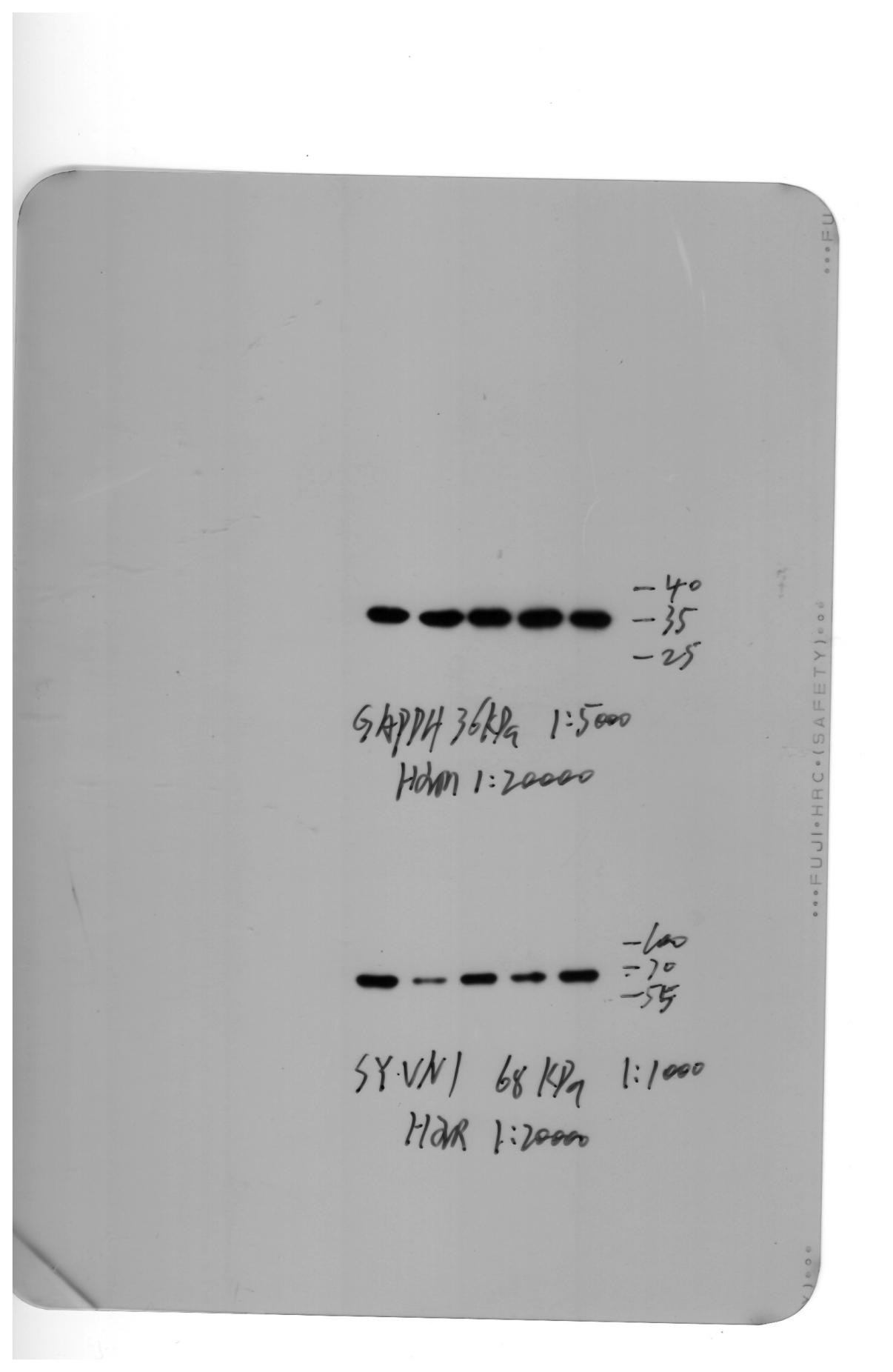


Figure 2A:


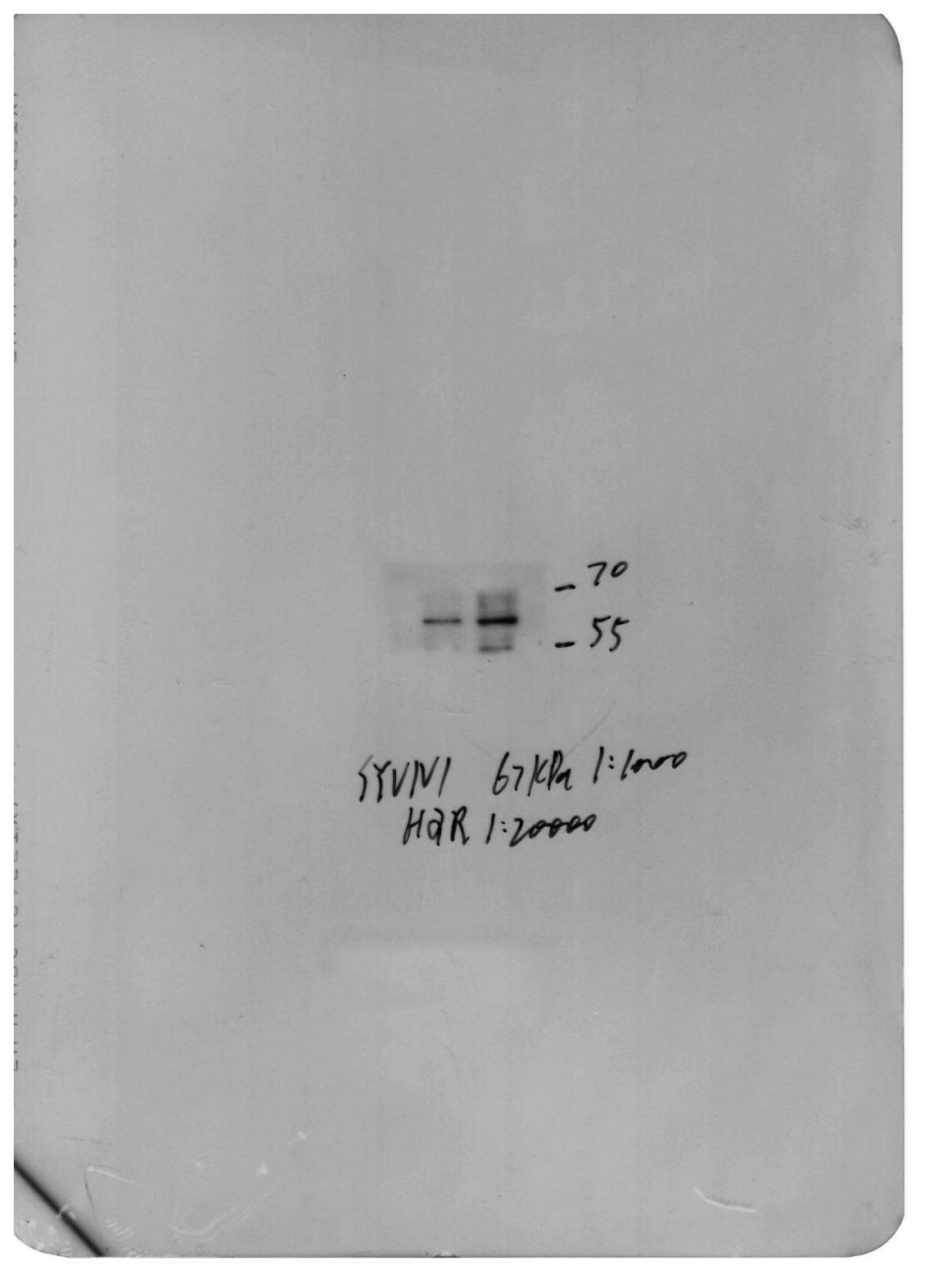


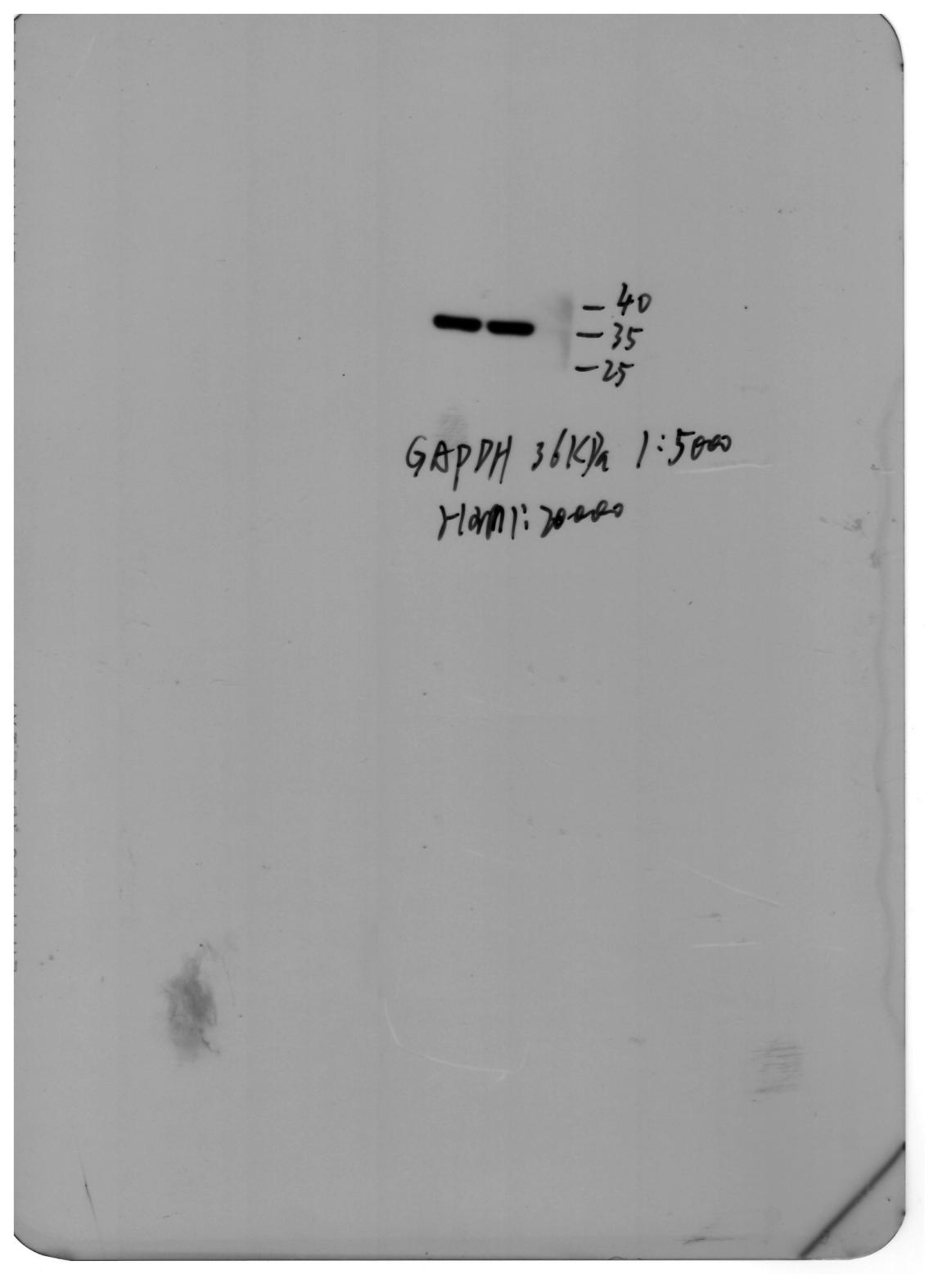


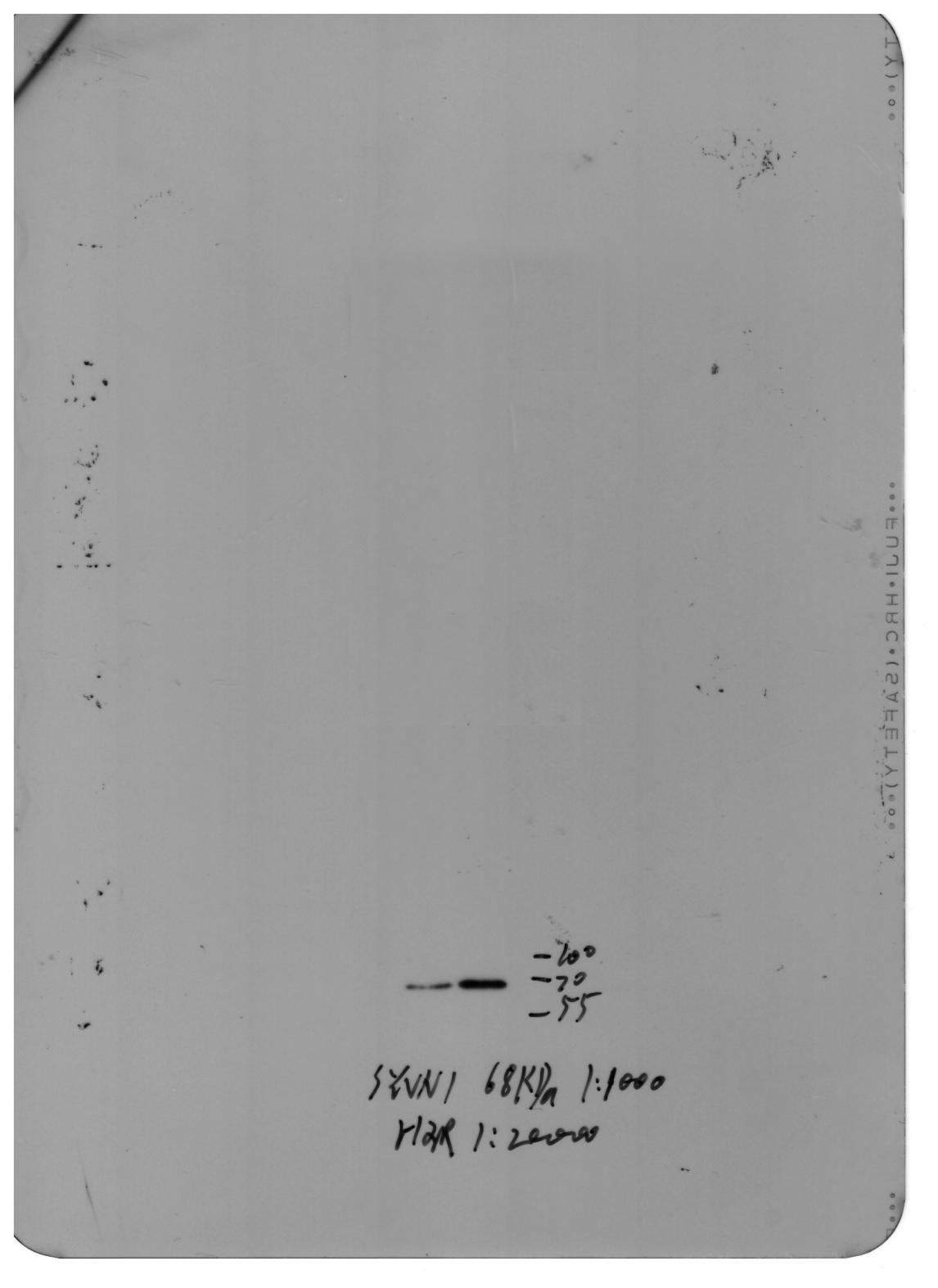


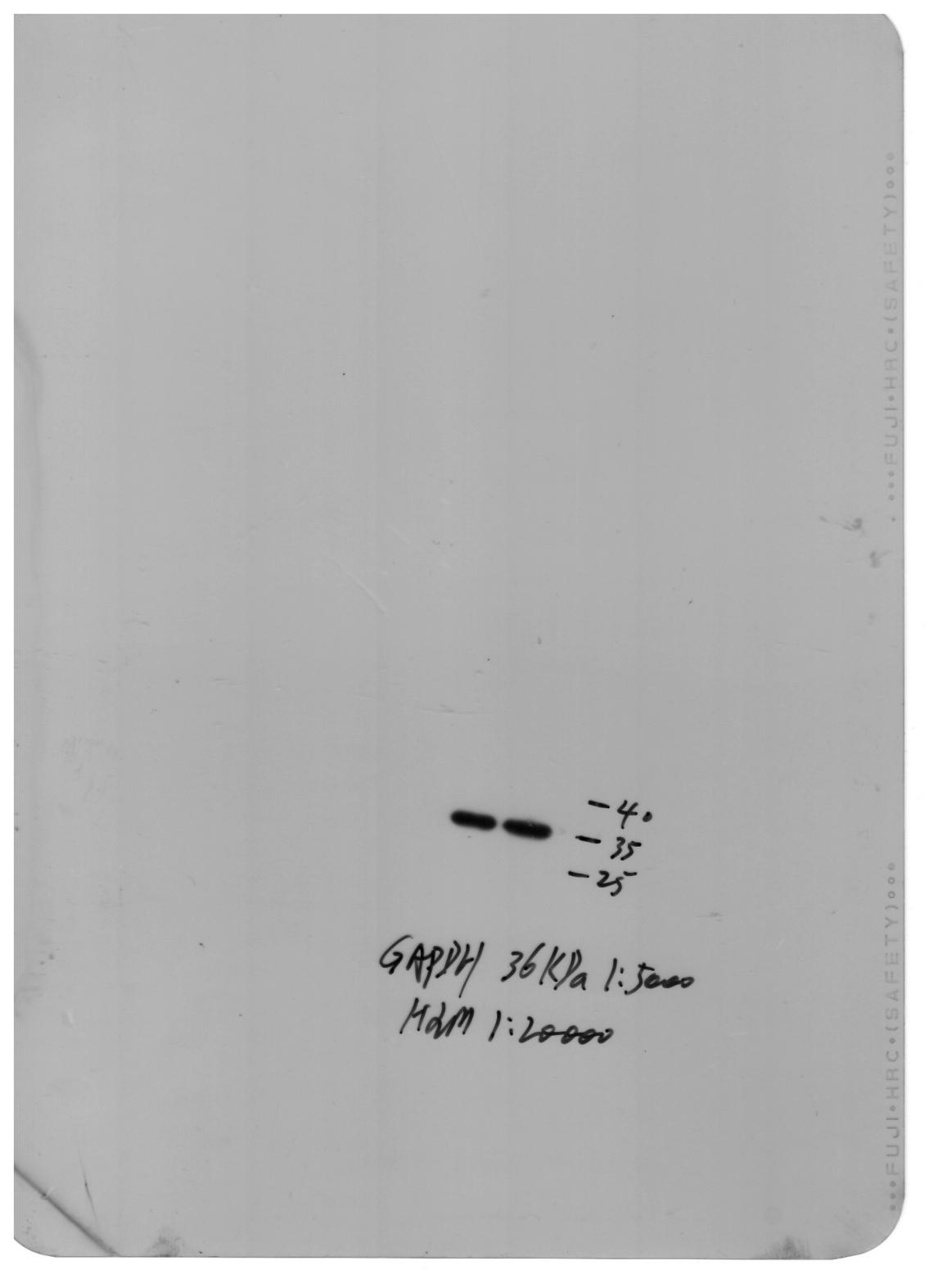


Figure 4B:


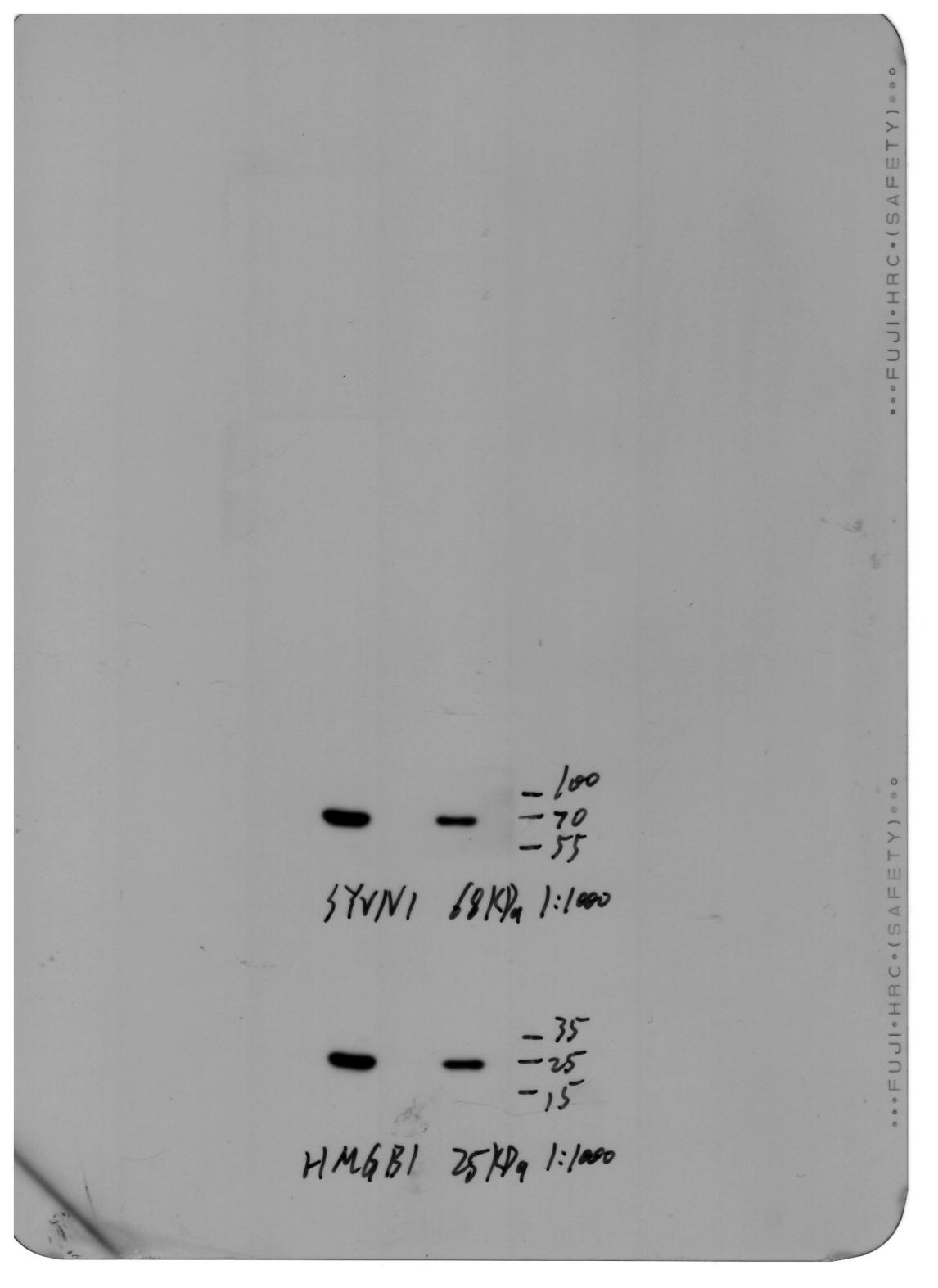


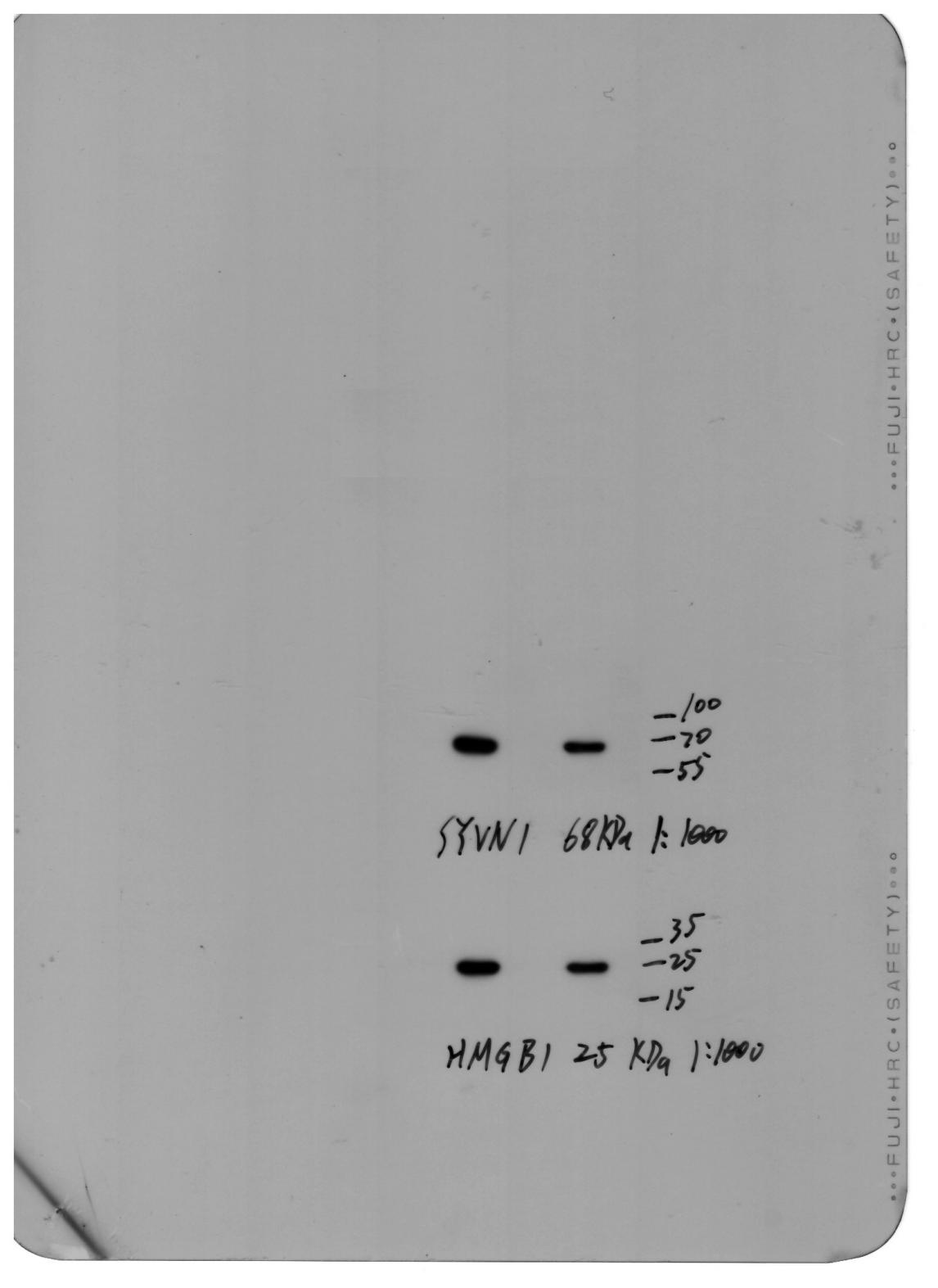


Figure 4C:


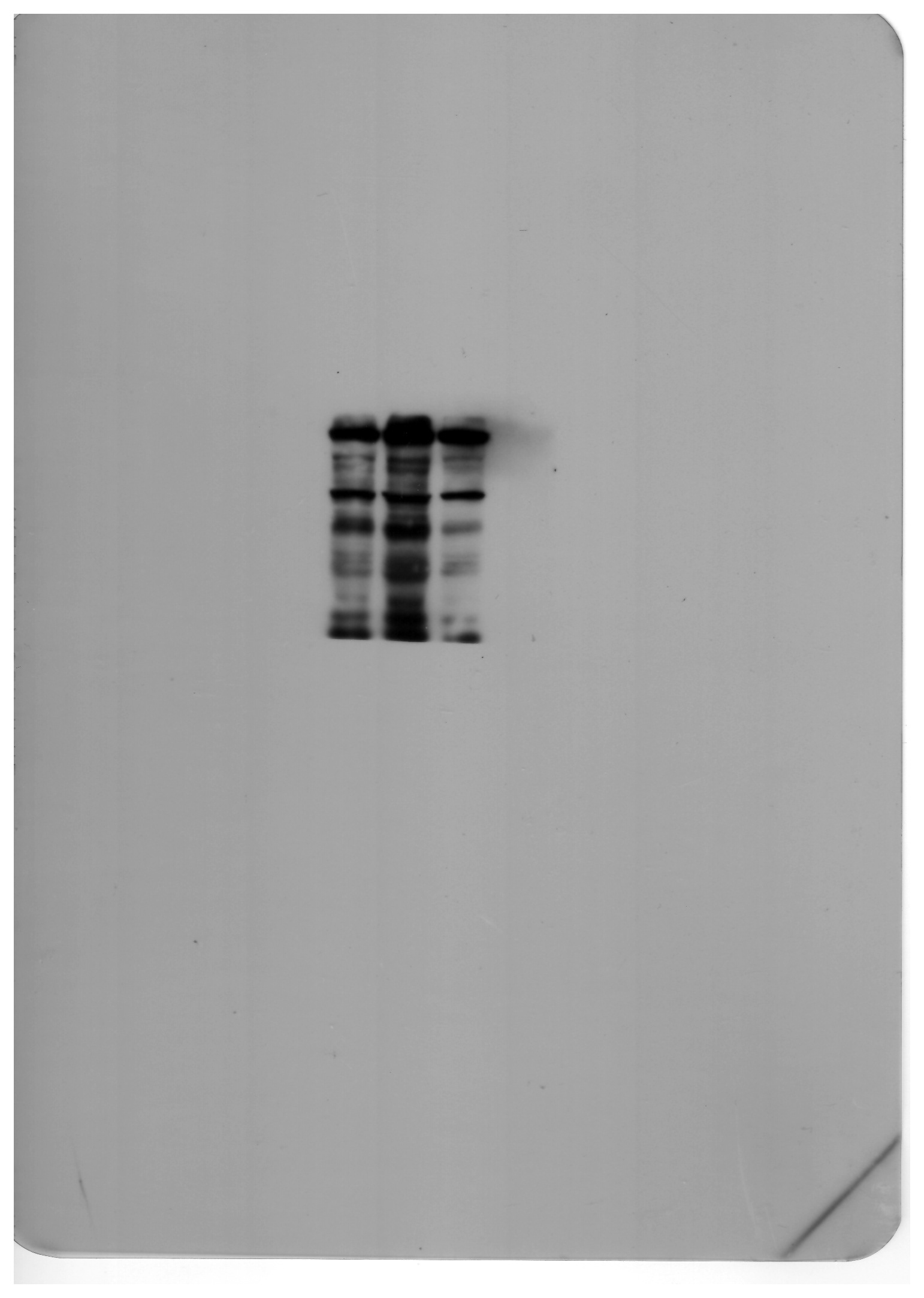


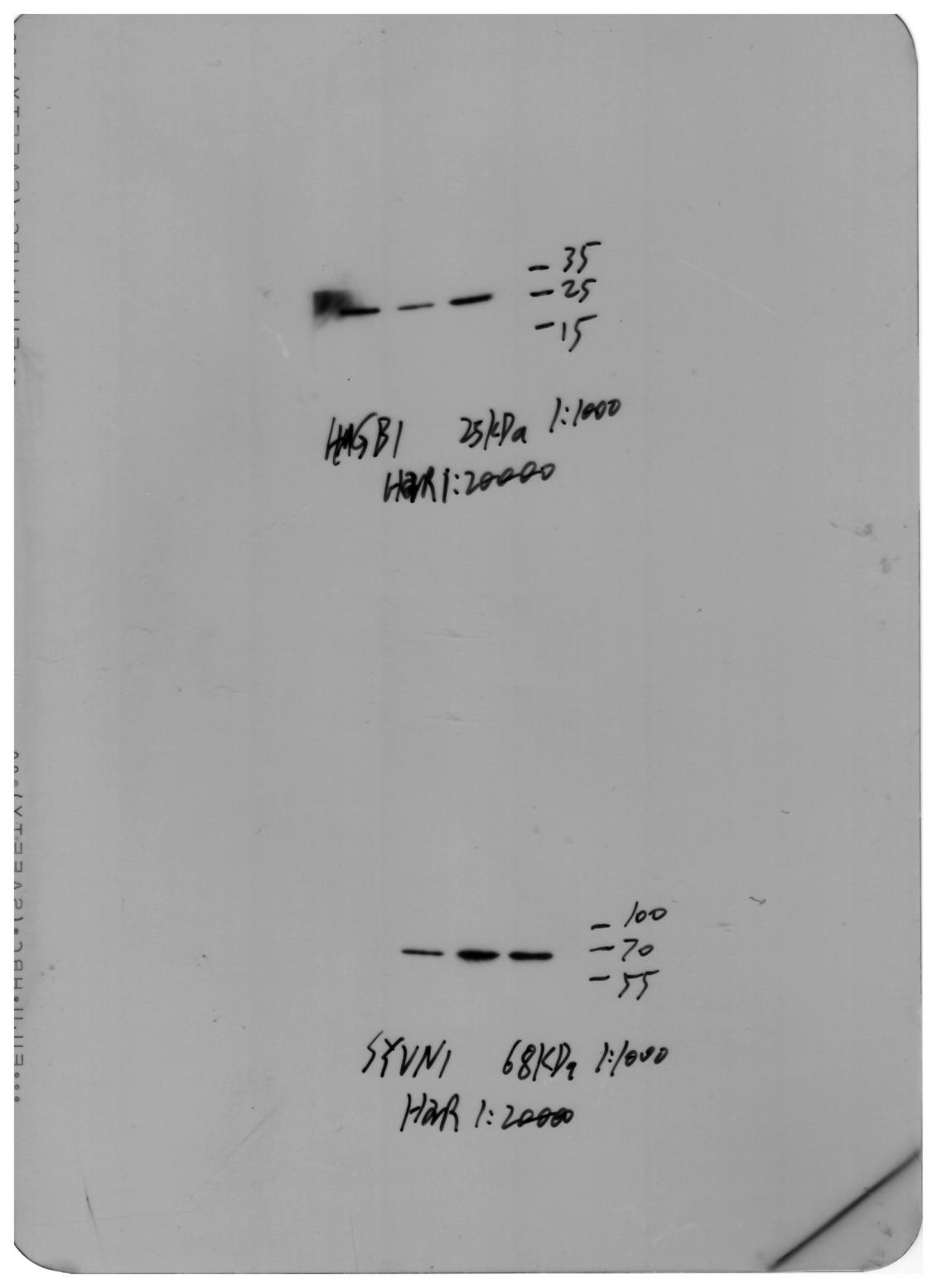

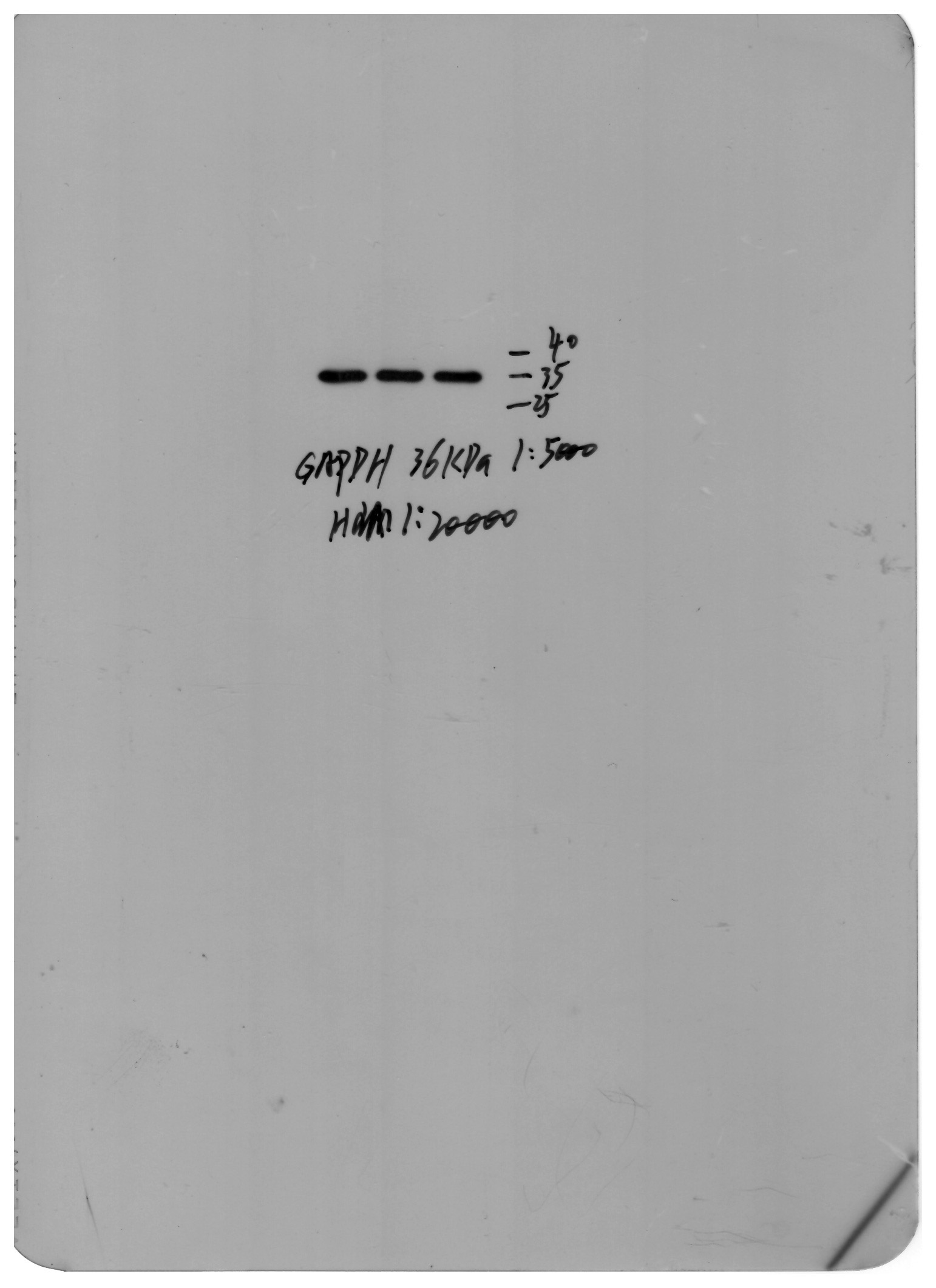


Figure 4D:


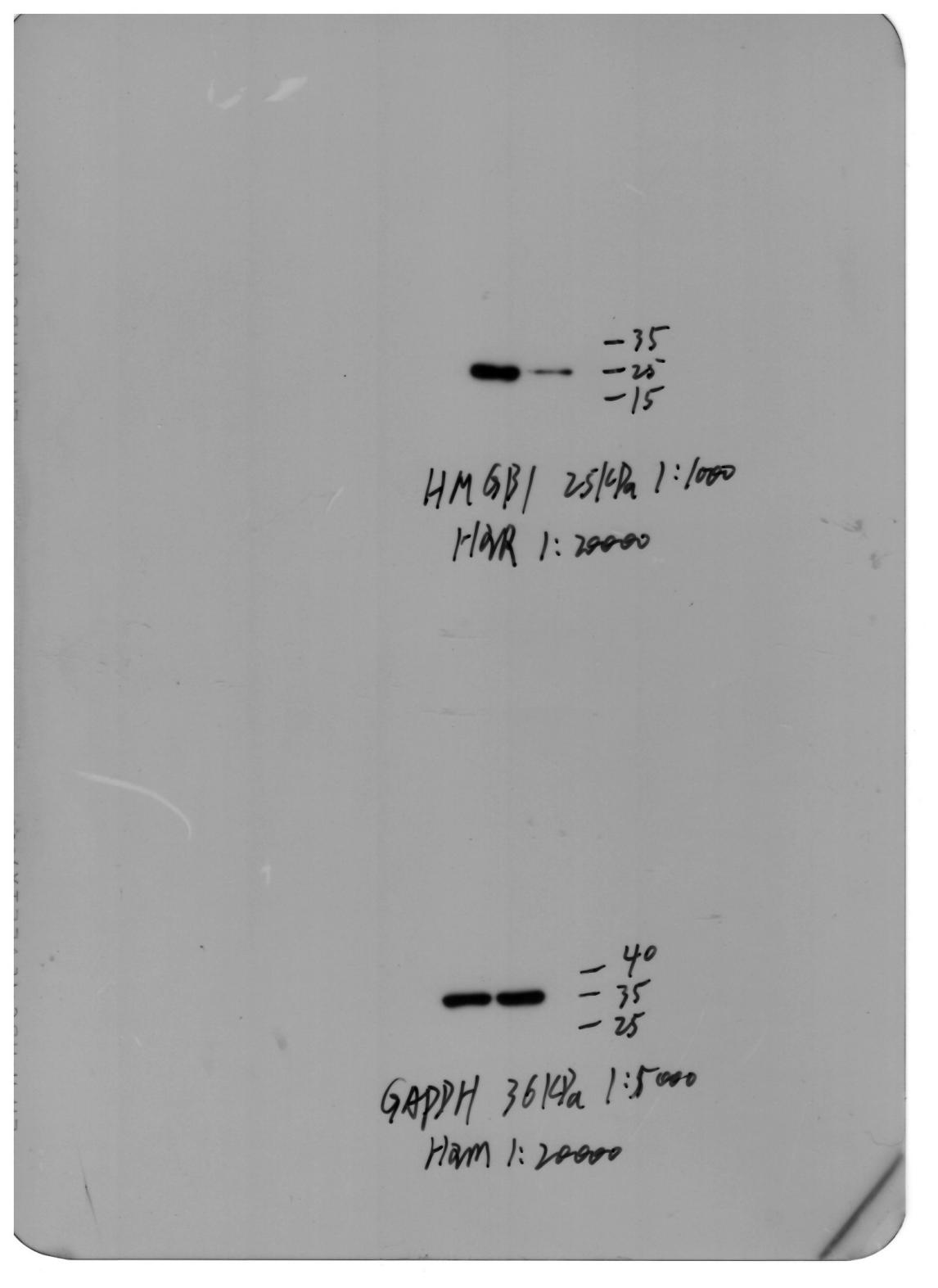


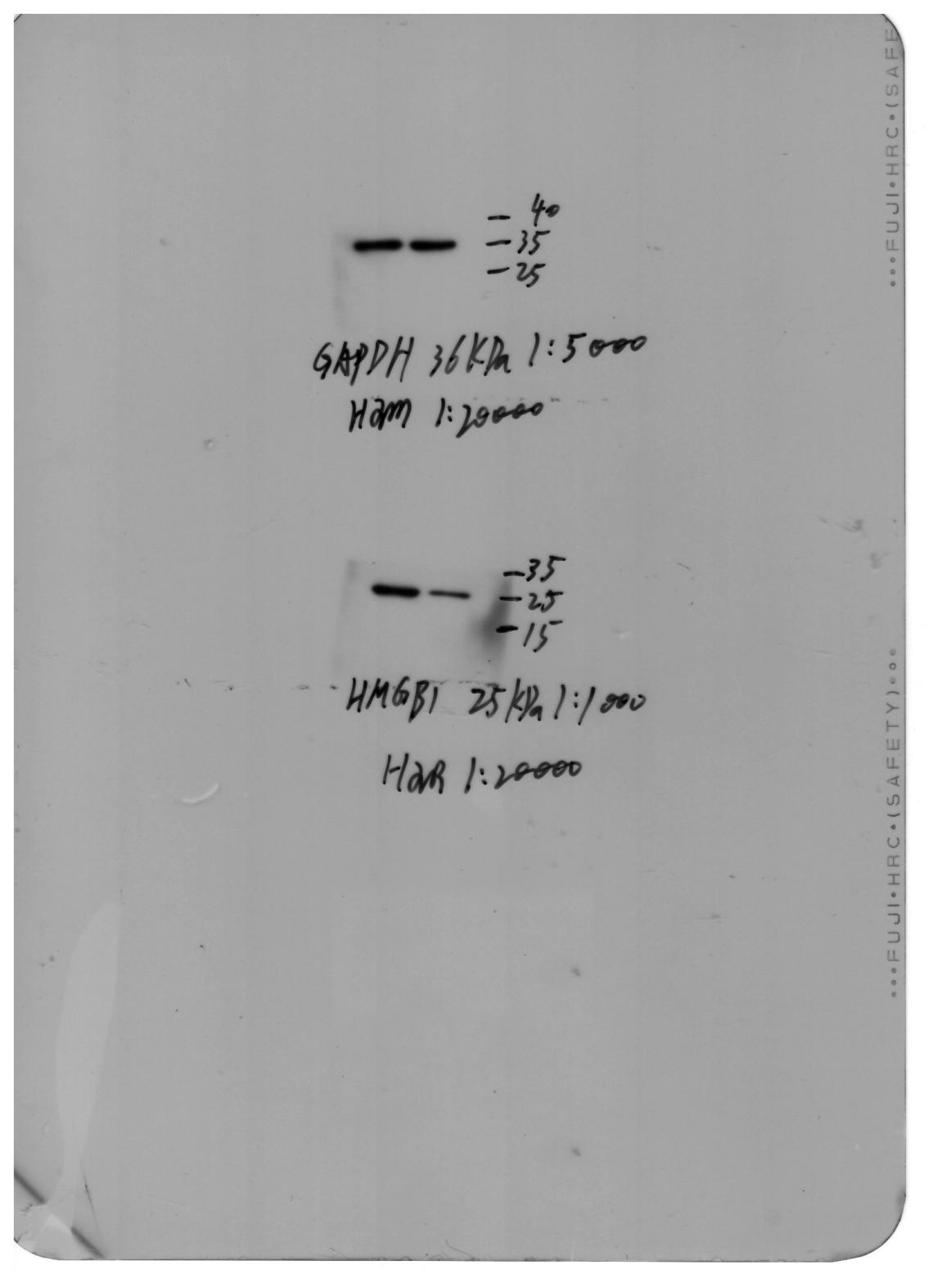


Figure 4E:


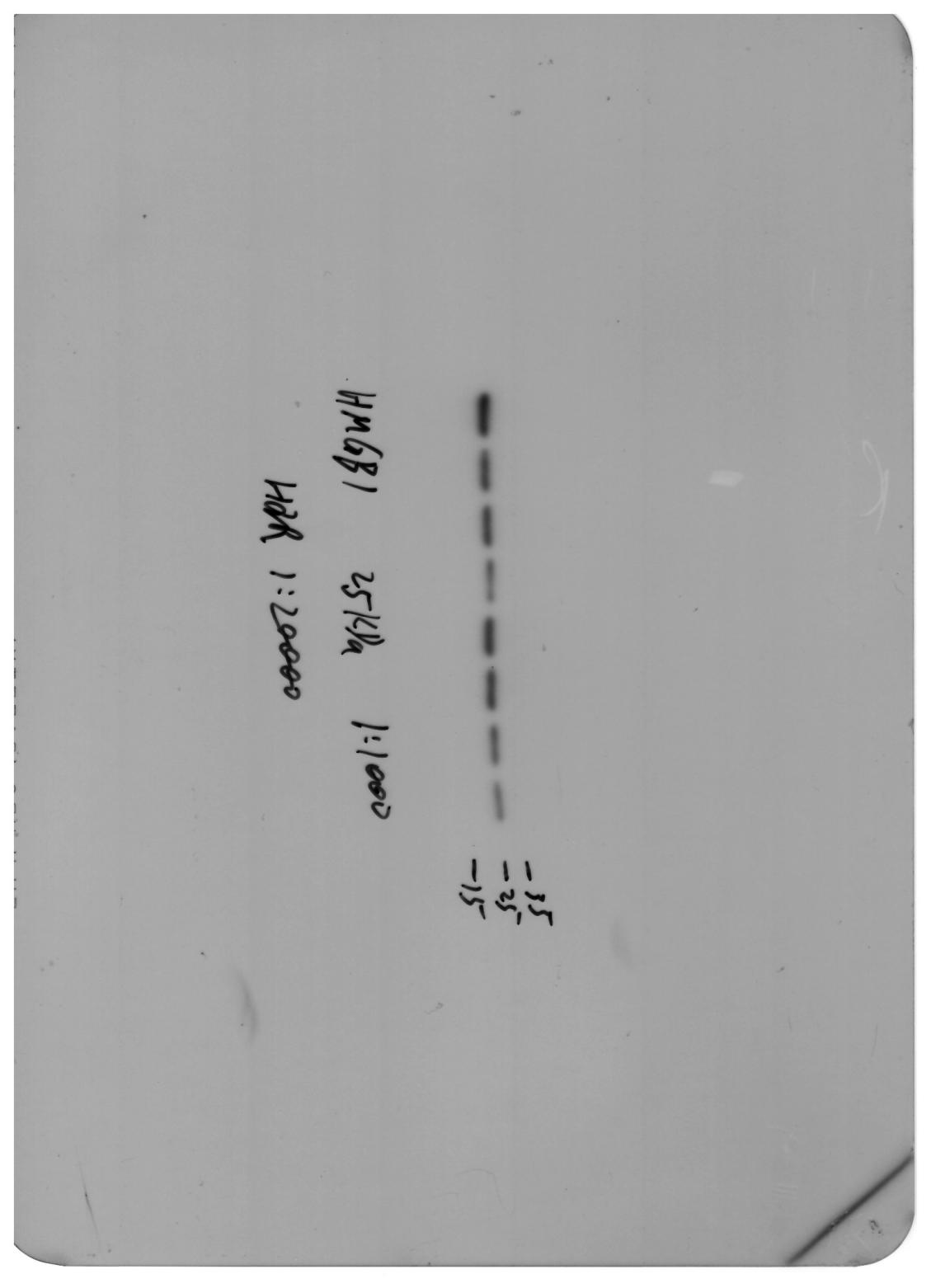


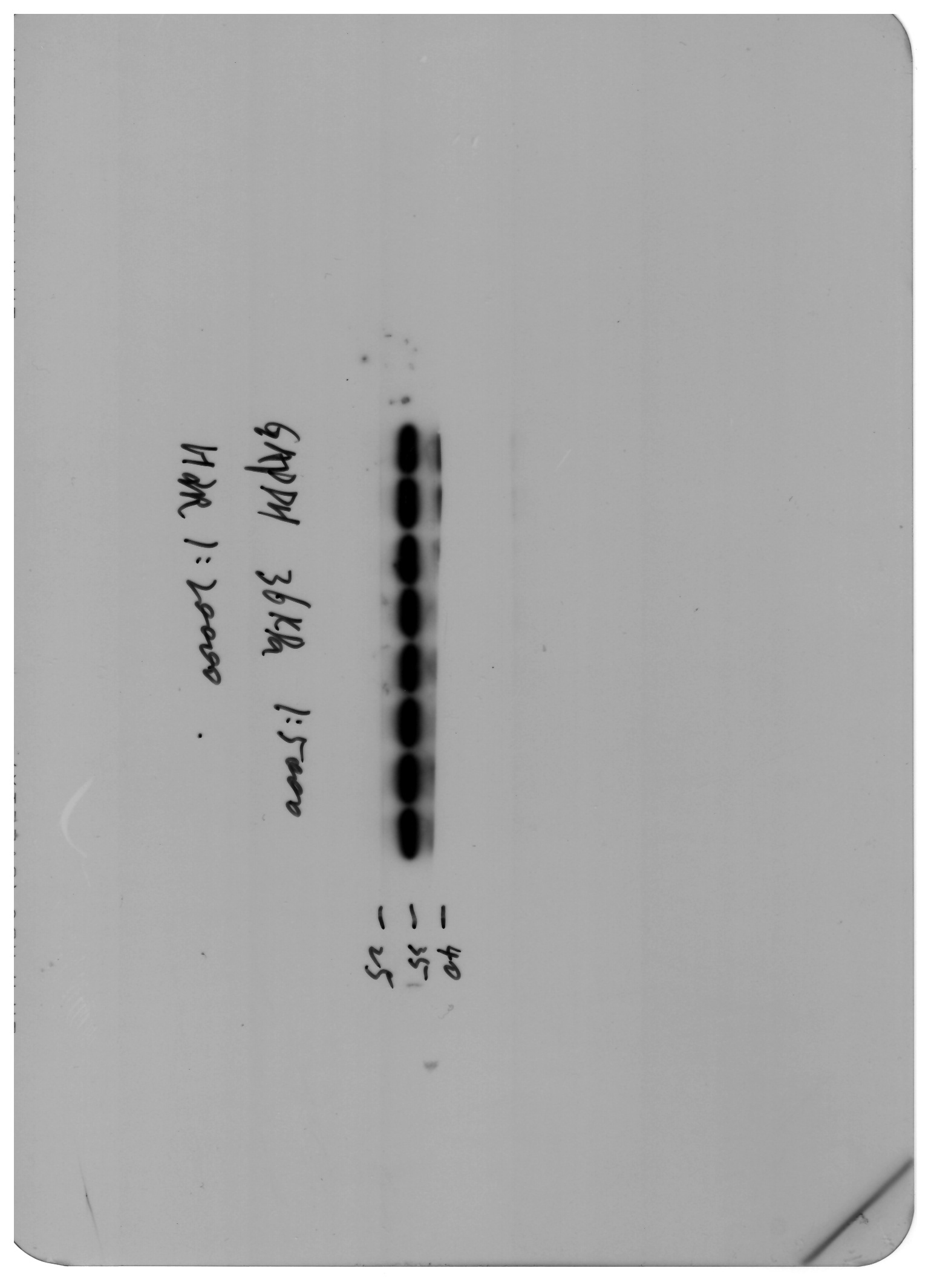


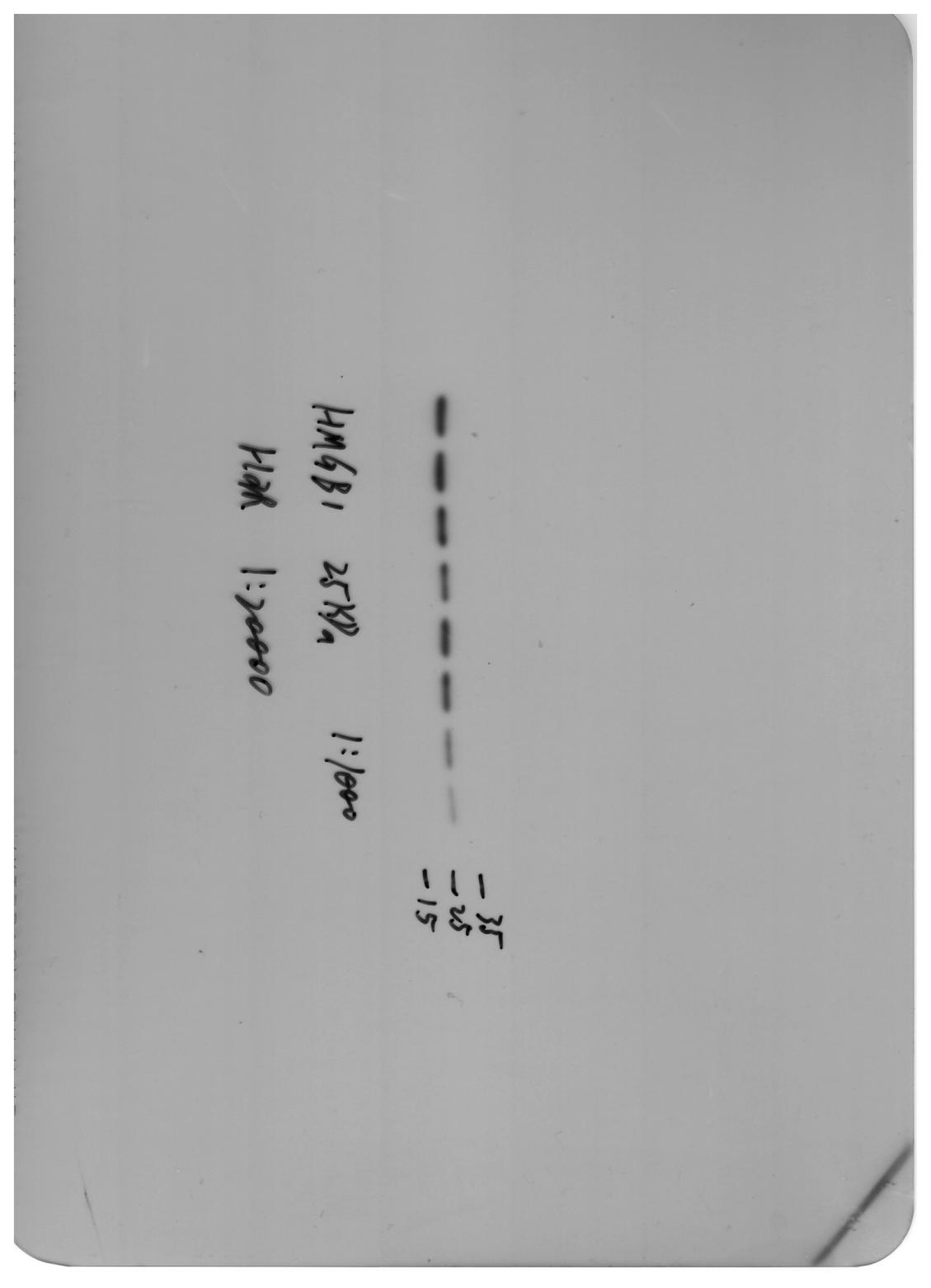


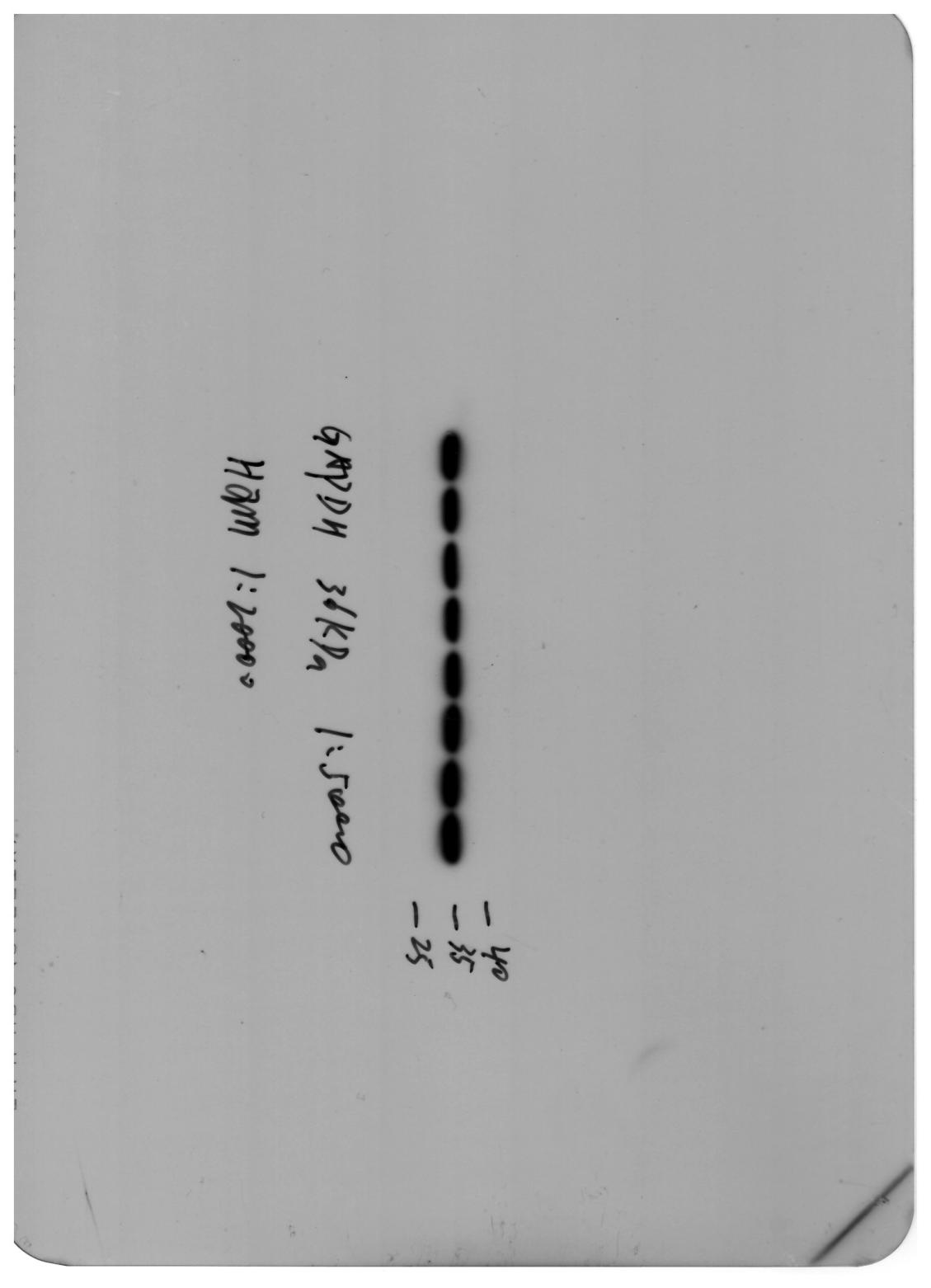

Supplement: Supplementary file 1 — Supplementary Material 1 [file 13008_2024_121_MOESM1_ESM.docx]
